# Sustainable Nanocomposite Films and Coatings for Meat Product Preservation: Recent Advances, Challenges, and Future Perspectives

**DOI:** 10.3390/foods15152632

**Published:** 2026-07-27

**Authors:** Wondemu Bogale Teseme, Shuai Wei, Jun Zhang, Shucheng Liu

**Affiliations:** 1 Guangdong Provincial Key Laboratory of Aquatic Product Processing and Safety, Guangdong Province Engineering Laboratory for Marine Biological Products, Guangdong Provincial Engineering Technology Research Center of Seafood, Key Laboratory of Advanced Processing of Aquatic Product of Guangdong Higher Education Institution, College of Food Science and Technology, Guangdong Ocean University, Zhanjiang 524088, China; wondemu.bogale@obu.edu.et (W.B.T.); weishuaiws@126.com (S.W.); 2 Department of Food Engineering, Institute of Technology, Oda Bultum University, Chiro 226, Ethiopia; 3 Collaborative Innovation Center of Seafood Deep Processing, Dalian Polytechnic University, Dalian 116034, China

**Keywords:** nanocomposite films, meat preservation, biodegradable packaging, antimicrobial coatings, shelf life extension

## Abstract

Meat and meat products are highly susceptible to microbial spoilage, lipid oxidation, moisture loss, discoloration, and sensory deterioration, creating a need for effective, safe, and sustainable packaging solutions. Although previous studies have investigated biodegradable polymers, nanomaterials, and active packaging systems separately, an integrated assessment connecting material design, preservation mechanisms, safety, sustainability, and commercial feasibility remains limited. This review addresses this gap by critically evaluating recent advances in biodegradable nanocomposite films and coatings for meat preservation. Current evidence demonstrates that the incorporation of nanoscale reinforcements and bioactive agents into biopolymer matrices can enhance their mechanical performance, gas and moisture barrier properties, antimicrobial activity, antioxidant capacity, and controlled release behavior. However, these advantages are strongly influenced by the nanofiller characteristics, concentration, dispersion, polymer-nanofiller interactions, food matrix composition, and storage conditions. Excessive nanomaterial incorporation may promote aggregation, induce structural defects, reduce flexibility, and increase migration concerns. Despite promising preservation outcomes, most available studies remain limited to laboratory-scale investigations, variable testing protocols, and insufficient validation under real commercial conditions. Key challenges hindering industrial adoption include nanoparticle migration, long-term safety assessment, regulatory uncertainty, production costs, consumer acceptance, and limited life-cycle evaluation. Future research should focus on safe-by-design formulations, standardized real-food testing, scalable manufacturing approaches, controlled-release technologies, and integrated assessments of preservation efficiency, safety, economic feasibility, and environmental sustainability. Overall, biodegradable nanocomposite packaging represents a promising approach for extending meat shelf life; however, successful commercialization requires balancing enhanced preservation performance with safety assurance and industrial practicality.

## 1. Introduction

The global food system is facing increasing challenges associated with population growth, climate change, resource limitations, and rising consumer demand for safe, nutritious, and shelf-stable food products. Global food demand is projected to increase substantially between 2010 and 2050, with estimates ranging from 35% to 56%, while climate change may further influence future food production and supply chains [1,2]. Moreover, food loss and waste remain major global concerns. One-third of all food produced worldwide is lost or wasted annually, resulting in considerable economic, environmental, and social impacts [3,4]. A significant proportion of these losses occur throughout production, transportation, storage, and retail due to microbial spoilage, oxidation, moisture migration, and quality deterioration [5].

Among food products, meat and meat-based foods are particularly susceptible to deterioration because of their high moisture content, abundant nutrients, and favorable conditions for microbial growth. These characteristics promote the proliferation of spoilage and pathogenic microorganisms, enzymatic reactions, lipid oxidation, protein oxidation, discoloration, off-flavor development, and texture deterioration during storage. Meat processing operations can further increase susceptibility to spoilage by removing natural protective barriers, such as skin structures and endogenous antimicrobial components [6]. Consequently, meat spoilage contributes substantially to economic losses, food waste generation, and environmental burdens. Spoilage-related losses in meat products have been reported to reach considerable levels during the production, distribution, storage, and marketing stages [7].

Packaging is a critical strategy for maintaining meat quality and extending shelf life by controlling exposure to oxygen, moisture, microorganisms, and external contaminants. Conventional petroleum-based plastic packaging materials remain dominant in the food industry because of their low cost, flexibility, lightweight nature, and excellent barrier properties. However, their limited biodegradability and persistent accumulation in terrestrial and aquatic environments have raised serious concerns regarding plastic pollution and long-term sustainability [8]. These environmental challenges have accelerated research toward renewable, biodegradable, and functional packaging alternatives capable of maintaining food safety while reducing ecological impacts.

Biopolymer-based films and coatings have emerged as promising alternatives to conventional plastics because of their renewable origin, biodegradability, and potential suitability for food-contact applications. Natural polymers, including starch, chitosan, cellulose, alginate, gelatin, soy protein, whey protein, and lipid-based materials, have been extensively investigated for biodegradable packaging development [8,9]. These materials can form continuous matrices capable of reducing moisture loss, limiting oxygen transfer, controlling microbial growth, and delaying oxidative deterioration in meat products. However, despite their environmental advantages, most single-component biopolymer films exhibit insufficient mechanical strength, high sensitivity to moisture, poor water vapor resistance, limited thermal stability, and inadequate barrier performance compared with those of conventional plastics [10]. These inherent limitations restrict their practical application under the high-moisture, refrigerated, and mechanically demanding conditions encountered in meat packaging.

To overcome these limitations, recent research has focused on developing multifunctional active biodegradable packaging systems by incorporating antimicrobial and antioxidant compounds into polymer matrices. Emerging strategies include combining multiple antimicrobial agents to achieve synergistic effects, incorporating compounds generally recognized as safe (GRAS) or already approved as food additives, and encapsulating bioactive substances within nano- or microcarriers to improve stability and provide controlled release. For example, Motelica et al. [11] demonstrated that a biodegradable hydroxyethyl cellulose-based packaging system containing zinc oxide nanoparticles and encapsulated cinnamon essential oil provided prolonged antimicrobial activity through controlled release mechanisms. Such approaches improve the efficiency of active packaging systems while minimizing the quantity of antimicrobial agents needed. Among biodegradable polymers, chitosan is particularly attractive because of its intrinsic antimicrobial properties [12]. Unlike starch, cellulose, alginate, and pectin, which act mainly as passive structural matrices, chitosan possesses positively charged amino groups that interact with negatively charged microbial cell membranes, resulting in membrane disruption, leakage of intracellular components, metal ion chelation, and interference with microbial metabolism [13]. Therefore, chitosan functions not only as a biodegradable film-forming material but also as an active antimicrobial component with strong synergistic potential when combined with nanoparticles, essential oils, bacteriocins, and plant-derived bioactive compounds.

Nanocomposite technology has further expanded the functional potential of biodegradable packaging materials by incorporating nanoscale fillers into polymer matrices. Common nanofillers investigated for food packaging applications include cellulose nanocrystals, nanoclays, zinc oxide nanoparticles, titanium dioxide nanoparticles, and silver nanoparticles. Owing to their physicochemical characteristics, particle size, concentration, dispersion behavior, and compatibility with the polymer matrix, nanofillers can enhance the mechanical strength, thermal stability, barrier properties, antimicrobial activity, and antioxidant performance. Improved interfacial interactions between nanofillers and polymer chains may create more compact structures that reduce gas and moisture permeability. However, excessive nanofiller loading or poor dispersion can promote aggregation, generate structural defects, reduce flexibility, and negatively affect overall packaging performance [14,15].

These multifunctional properties are particularly valuable for meat preservation because spoilage occurs through multiple interacting mechanisms rather than a single deterioration pathway. Microbial growth, lipid oxidation, protein oxidation, moisture migration, color degradation, and sensory changes simultaneously influence meat quality during storage. Recent studies have demonstrated that nanocomposite films and coatings can extend the shelf life of fresh red meat, poultry, processed meat, seafood, and ready-to-eat meat products by inhibiting microbial proliferation, delaying oxidation, maintaining color stability, and preserving texture quality [15,16]. In addition, some advanced systems provide intelligent or responsive functions, including controlled antimicrobial release, antioxidant protection, and freshness monitoring.

Despite these advances, several challenges continue to limit the transition of nanocomposite biodegradable packaging from laboratory research to commercial application. Nanoparticle migration into food remains an important safety concern; however, migration behavior is highly dependent on the nanomaterial type, particle characteristics, polymer structure, concentration, food composition, and storage conditions [17]. Furthermore, uncertainties remain regarding long-term toxicity, environmental fate, and potential ecological impacts after disposal. In addition to safety issues, reported improvements in nanocomposite packaging performance often vary considerably among studies [18]. Differences in polymer matrices, nanofiller compositions, particle sizes, processing techniques, testing methods, and food models make direct comparisons challenging. Although many studies have demonstrated promising antimicrobial, mechanical, and barrier improvements under controlled laboratory conditions, these benefits may not always be maintained in real food systems because of complex interactions among packaging materials, microorganisms, food components, and storage environments.

Moreover, the industrial application of biodegradable nanocomposite packaging remains limited despite extensive academic research. Challenges associated with large-scale manufacturing, consistent nanofiller dispersion, processing compatibility with existing packaging technologies, production cost, regulatory approval, consumer acceptance, and end-of-life management continue to restrict commercialization [19]. Importantly, the sustainability of nanocomposite packaging cannot be evaluated solely on the basis of the biodegradability of the polymer matrix. A comprehensive assessment should also consider nanofiller production, energy consumption, environmental release, recyclability, and life-cycle impacts. Therefore, future development requires an integrated approach that balances preservation efficiency, safety, economic feasibility, and environmental sustainability.

**Figure 1:**
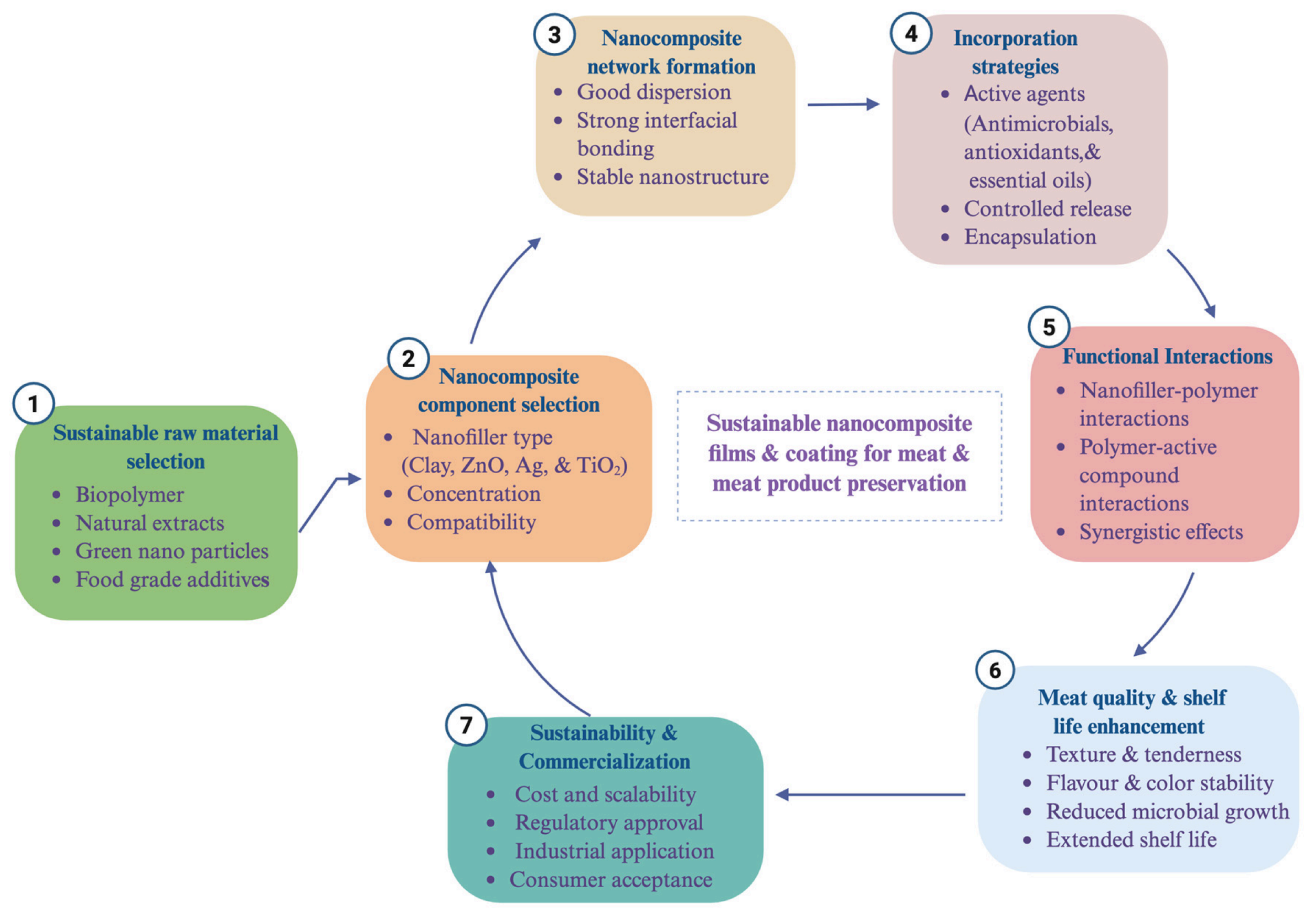
Conceptual framework for sustainable nanocomposite packaging development: from material selection to predictive design and commercialization. The schematic illustrates the sequential stages of (1) sustainable raw material selection, (2) nanocomposite component optimization, (3) nanostructure formation through dispersion and interfacial interactions, (4) incorporation of active agents with controlled release strategies, (5) functional interactions among polymers, nanoparticles, and bioactive compounds, (6) improvement of meat quality and shelf life, and (7) sustainability and commercialization considerations.

Previous researchers have focused mainly on biodegradable films, antimicrobial packaging systems, or individual nanomaterial applications. However, a comprehensive critical assessment of sustainable nanocomposite films and coatings specifically designed for meat preservation, integrating material mechanisms, preservation performance, safety evaluation, regulatory considerations, sustainability aspects, and industrial feasibility, remains limited. Therefore, this review provides an updated and critical analysis of sustainable nanocomposite films and coatings for meat product preservation. This review discusses the mechanisms of meat spoilage, limitations of conventional packaging, advances in biodegradable polymer systems, and the role of nanofillers in improving functional properties. Applications in fresh red meat, poultry, processed meat, seafood, and ready-to-eat meat products are critically evaluated, followed by discussions on safety concerns, regulatory challenges, sustainability considerations, and future research directions. The main contribution of this review is the establishment of an integrated perspective connecting material design, preservation mechanisms, safety assessment, and commercialization pathways for next-generation sustainable meat packaging systems (Figure 1).

### Review Methodology

A literature search was conducted to comprehensively review sustainable nanocomposite films and coatings for meat product preservation. The following electronic databases were consulted: Scopus, Web of Science, PubMed, Google Scholar, and ScienceDirect. The search covered the period from 2017 to 2026, capturing recent advances while including foundational studies where necessary. The main search combinations included: (“nanocomposite film” OR “nanocomposite coating”) AND (“meat” OR “meat products” OR “fresh meat” OR “poultry” OR “processed meat” OR “seafood” OR “ready-to-eat meat”) AND (“preservation” OR “shelf life” OR “spoilage”); (“biodegradable packaging” OR “edible film” OR “active packaging”) AND (“meat preservation” OR “meat quality”); (“chitosan” OR “starch” OR “cellulose” OR “pullulan” OR “alginate” OR “gelatin” OR “soy protein” OR “whey protein” OR “lipid-based packaging film”) AND (“film” OR “coating” OR “nanocomposite packaging”) AND (“meat” OR “food preservation”); (“ZnO nanoparticles” OR “TiO_2_ nanoparticles” OR “silver nanoparticles” OR “nanoclay” OR “cellulose nanocrystals”) AND (“food packaging” OR “meat packaging”); (“antimicrobial activity” OR “antioxidant activity”) AND (“nanocomposite packaging” OR “active packaging”) AND “meat”; (“nanocomposite characterization” AND (SEM OR FTIR OR XRD OR TGA OR mechanical properties OR barrier properties)); and (“nanoparticle migration” OR “toxicity” OR “regulatory safety”) AND (“food contact materials” OR “food packaging”). Only articles published in English were considered. The reference lists of the selected articles were also manually searched to identify additional relevant studies. The review follows the principles of systematic review methodology to ensure comprehensive coverage and reproducibility, although it is not designed as a formal systematic review or meta-analysis.

## 2. Meat Spoilage Mechanisms and Preservation Requirements

**Figure 2:**
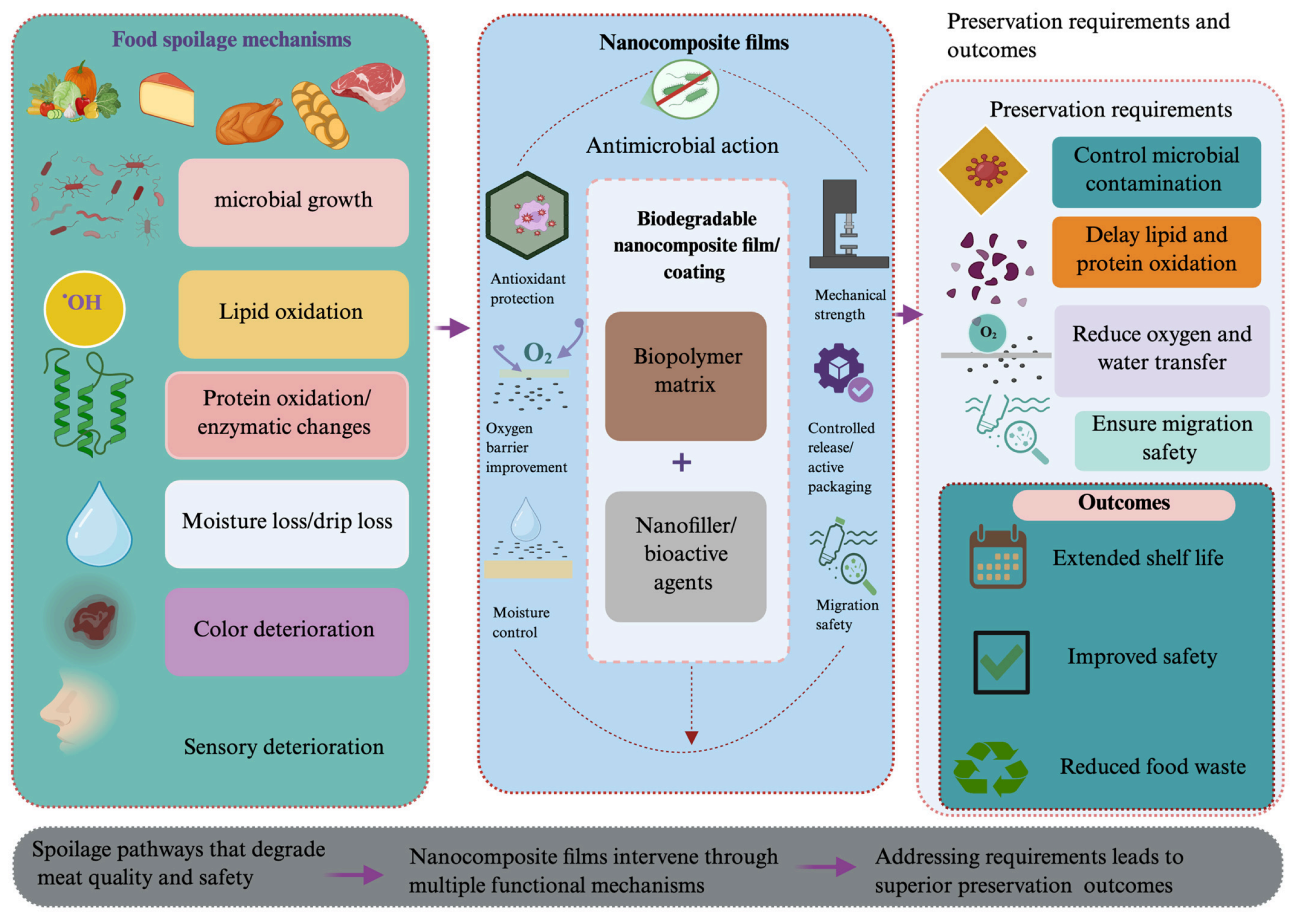
Meat spoilage mechanisms and preservation requirements. Meat spoilage pathways and nanocomposite-based preservation strategies for quality and shelf-life improvement. The left panel illustrates major meat spoilage mechanisms, including microbial growth, lipid oxidation, protein oxidation, moisture loss, color deterioration, and sensory degradation. The middle panel presents the structure and functional mechanisms of nanocomposite films, including biopolymer matrices, nanofillers/bioactive agents, antimicrobial activity, antioxidant protection, barrier properties, and controlled release functions. The right panel summarizes preservation requirements and outcomes, including microbial control, oxidation delay, reduced oxygen and water transfer, migration safety, extended shelf life, improved safety, and reduced food waste.

Meat is a very high-risk food for spoilage due to its high moisture content and protein composition and its lipid, mineral, vitamin and nutrient contents, which support both microbiological development and biochemical reactions. Post-slaughter cessation of blood flow leads to postmortem transformations, including anaerobic glycolysis, a reduction in pH levels, exhaustion of cellular energy stores, protein breakdown, structural alterations in skeletal muscle tissue and other types of damage [20,21]. The subsequent steps in producing processed meats such as butchering or cutting, grating or chopping, packaging/processing, distribution/transiting and retail/handling all result in an increased surface area of the meat product exposed to both microorganisms and oxygen along with additional environmental stresses. Thus, the combined effects of microorganism proliferation, oxidation, enzymatic degradation, moisture migration, and coloration lead to progressive decreases in the sensory characteristics, nutritional value and consumer acceptance of meat products (Figure 2).

Microbial activity is the main reason for meat deterioration. Contamination can occur in many ways during slaughtering, while internal organs can be removed when the organism is cut or ground, during transportation, when it is displayed by a retailer, and when it is handled because of the presence of microorganisms from animal hides, the gastrointestinal tract, water used in processing, equipment, air, workers and contact with other foods. Both the original microbial load and physicochemical conditions, such as temperature, pH, water activity, oxygen availability, relative humidity, storage time and interactions among microbes affect the growth of spoiled microbial flora. Under aerobic refrigerated conditions, psychrotrophic bacteria, particularly *Pseudomonas* species, commonly dominate because of their ability to grow at low temperatures [21].

In addition to *Pseudomonas* spp., other major spoilage microorganisms associated with meat include *Brochothrix thermosphacta*, *Enterobacteriaceae*, lactic acid bacteria, yeast and molds. However, microbial communities are dependent on the meat type and storage conditions [22,23]. When stored, microbes metabolize carbohydrates, proteins, peptides and lipids to produce organic acids, alcohols, aldehydes, ketones, ammonia, biogenic amines, sulfurcontaining compounds and additional metabolites that contribute to odor production, slime formation, gas generation, color changes and texture degradation [24,25,26].

Chemical oxidation is the second most important factor for the spoilage of meat. Lipid oxidation is an oxidative reaction that leads to the generation of lipid radicals, hydroperoxides and secondary lipid oxidation products, aldehydes and ketones due to light, heat, reactive oxygen species and transition metals. These products lead to off-flavors, unpleasant odors, nutrient degradation, and shelf life shortening. Lipid oxidation can be significantly affected by fatty acid composition, available oxygen, temperature, processing damage, salt concentration, and the presence of heme and non-heme iron [27,28]. Furthermore, lipid derived radical species and reactive aldehydes may cause oxidative degradation of both proteins and pigments which could increase the rates at which the oxidative degradation of proteins occurs. The oxidative degradation of proteins is defined as the modification of amino acids, generation of carbonyls, loss of sulfhydryl groups, fragmentation of protein molecules, aggregation and crosslinking of protein molecules which results in less soluble protein products with a lower ability to hold onto water, tougher textures, and less desirable nutritional and functionality [29,30].

Moisture migration as well as structural modifications can result in a reduction in meat quality. A decrease in postmortem pH, denaturation of proteins, contraction of myofibrils, and decreased ability for water retention facilitate moisture migration from an intracellular space to an extracellular space. The increased moisture migration results in the loss of juice via drip loss and subsequent decreases in juiciness. These changes are accelerated when freezing and thawing occur because the development of ice crystals within the tissue disrupts membranes and causes irreparable structural damage. Each cycle of freezing and thawing increases the number of sites on proteins and lipids exposed to oxygen, enzymes, and pro-oxidant compounds which promotes both microbiological and oxidative degradation [31,32]. In addition, color degradation primarily results from myoglobin oxidation and the conversion of ferrous myoglobin to ferric metmyoglobin, resulting in undesirable browning. Myoglobin oxidation is influenced by oxygen availability, temperature, pH, antioxidant status, lipid oxidation, microbial growth and time in storage [33,34]. Overall, spoilage of meat is an extremely complex and multifactorial process. There are many interdependent processes involved in the spoilage of meat. Lipid and protein breakdown by microbial enzymes occurs simultaneously with the promotion of lipid breakdown into compounds that oxidize proteins and pigments. Moisture loss occurs concurrently with the inhibition of microbial growth and structural damage from enzymatic breakdown or physical damage promotes chemical reactions. The scientific understanding of each individual mechanism of spoilage has led to the development of multifunctional packaging materials that inhibit microbial growth, oxidative reactions, and moisture transfer [20,35]. The major spoilage pathways, their effects on meat quality, and related preservation requirements are summarized in Table 1.

## 3. Sustainable Materials for Nanocomposite Films and Coatings

**Figure 3:**
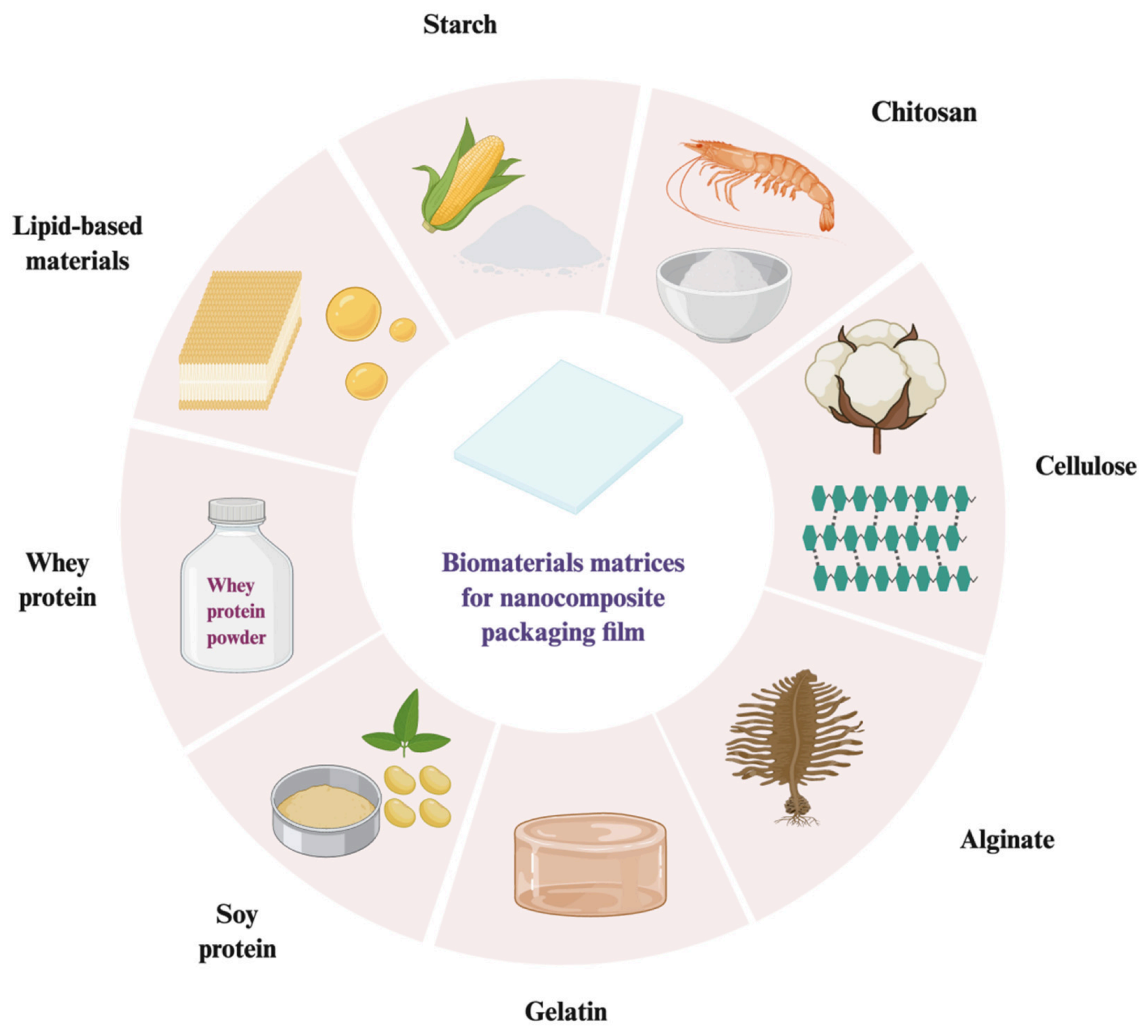
Biopolymer matrices for nanocomposite packaging films. The figure presents major biopolymer and lipid-based materials used as film-forming matrices, including starch, chitosan, cellulose, alginate, gelatin, soy protein, whey protein, and lipid-based materials, which provide the structural foundation for incorporating nanofillers and developing sustainable active packaging films.

Biopolymer films alone often do not fully meet the mechanical, thermal, and barrier requirements of meat packaging. The incorporation of nanofillers can address these limitations by improving the film structure and reducing gas and moisture permeability (Figure 3). Nanofillers such as nanoclays, nanocellulose, ZnO, TiO_2_, Ag nanoparticles, and essential oil nanoparticles can create a tortuous diffusion pathway, making it more difficult for oxygen and water vapor to pass through the film (Table 2). This phenomenon improves barrier performance and may also enhance antimicrobial and antioxidant activity, which is important for reducing spoilage and extending the shelf life of meat products [47].

### 3.1. Availability, Commercial Importance, and Research Trends of Sustainable Biopolymers

The use of biodegradable packaging products at a mass level for industrial purposes is determined primarily through the cost of manufacturing, cost of materials, processing needs and compatibility with current packaging processes [48]. Cellulose is an abundantly available, naturally occurring renewable polymer that has considerable industrial benefit because of its ease of access and well-established processing methods. A wide range of cellulose based materials are being utilized today in paper, paper board, regenerated cellulose film coatings and fiber-based packaging applications [49]. Starch is also very cheaply obtainable and economically viable as a biopolymer, as it may be derived from an abundance of food grade agricultural crops including corn, wheat, potatoes and cassava. Owing to their compatibility with thermoplastic processing and their relatively low cost, starch-based films represent one of the commercially viable options for biodegradable packaging materials [50,51,52]. In contrast, the availability and production pathways of other biopolymers may limit their industrial adoption. Chitosan is obtained mainly from crustacean shell waste generated by seafood-processing industries, providing an opportunity for marine byproduct valorization. However, its production requires several processing steps, including demineralization, deproteinization, deacetylation, and purification, which increase chemical consumption, energy requirements, and production costs [53,54]. Similarly, alginate is extracted mainly from brown seaweed and requires extraction and purification processes that influence production costs and material consistency [55]. Protein-based polymers based on food byproducts and agricultural products represent a renewable alternative for packaging applications, but limitations in the scale-up of these protein-based polymers include purification needs and the need for drying as well as potential allergens and water sensitivity [56]. Although pullulan has very good transparency, good oxygen barrier properties, and good film-forming ability, it is produced via the use of microorganisms that ferment starch-derived substrate material [57]. The need to control fermentation, remove pigments, and purify the product after fermentation contributes to higher production costs than those of cellulose- or starch-based products [58,59].

**Figure 4:**
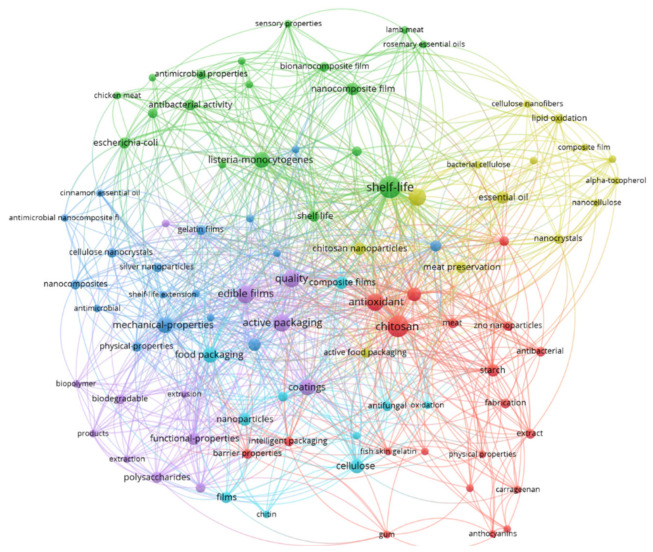
Keyword co-occurrence network of research trends in sustainable nanocomposite films and coatings for meat product preservation, generated using VOSviewer (Version 1.6.2, Netherlands). The node size represents keyword occurrence frequency, link thickness indicates co-occurrence strength, and different colors represent distinct thematic clusters.

Research trends reflect differences in both scientific interest and technological maturity among biodegradable polymers used for sustainable food packaging. Figure 4 presents a keyword co-occurrence analysis, highlighting chitosan, starch, and cellulose-based materials as the most extensively investigated biopolymer platforms. Chitosan has attracted the greatest amount of research attention because of its intrinsic antimicrobial activity, biodegradability, excellent film-forming ability, and compatibility with active packaging systems, making it one of the most widely studied materials for antimicrobial films and edible coatings. Starch-based materials also demonstrate high research prominence owing to their low cost, abundant availability, renewability, and suitability as biodegradable matrices for food packaging applications. Similarly, cellulose and its nanostructured derivatives, particularly nanocellulose, have emerged as key research areas because of their high mechanical strength, large aspect ratio, and remarkable ability to improve the barrier and reinforcement properties of nanocomposite films. In contrast, other biopolymers, including alginate, gelatin, soy protein, whey protein, lipid-based materials, and pullulan, have received comparatively less attention but represent rapidly expanding research areas, particularly edible coatings, multifunctional packaging, and hybrid composite systems. Overall, the keyword distribution indicates a shift from conventional biodegradable materials toward multifunctional and nanostructured biopolymer systems capable of simultaneously enhancing food preservation, extending shelf life, and reducing environmental impacts.

The global packaging sector continues to expand due to population growth, increasing consumption of packaged foods, and increasing demand for sustainable alternatives. The global food-packaging market was valued at USD 533.22 billion in 2025 and is projected to experience substantial growth in the coming years [60]. However, conventional petroleum-based plastics remain dominant because of their low production cost, excellent moisture resistance, mechanical strength, and well-established manufacturing infrastructure. Although biobased packaging materials are gaining increasing attention, their current market contribution remains limited due to challenges associated with cost competitiveness, scalability, barrier performance, and processing compatibility [61].

Among the currently investigated sustainable polymers, cellulose has the highest industrial readiness because of its abundance, established processing technologies, and current integration into commercial packaging applications. Its derivatives are already integrated into commercial paper-based packaging, coatings, and fiber-reinforced materials [62]. Starch also represents one of the most commercially promising biodegradable polymers because of its low cost, availability, and compatibility with existing thermoplastic processing technologies [63]. In comparison, chitosan, alginate, protein-based polymers, and pullulan remain largely concentrated in research, pilot-scale production, and specialized high-value applications. Nevertheless, these materials provide unique functional advantages, including antimicrobial activity, antioxidant capacity, film transparency, and compatibility with bioactive compounds, making them attractive candidates for advanced active packaging systems. Overall, cellulose has the highest industrial readiness because of its abundant supply and established infrastructure, whereas starch provides an excellent balance between availability, cost, and processability [64]. Chitosan remains one of the most promising polymers for active packaging applications because of its intrinsic antimicrobial functionality and potential for valorizing marine processing waste. Pullulan and protein-based polymers offer valuable functional properties but require further improvements in production economics and scalability.

**Table 2:** Biopolymer-based nanocomposite films, materials, nanofillers, preparation methods, and functional properties.

Category	Biopolymer Material	Nanoparticles/Nanofillers	Preparation Method	Key Functional Properties	Effect on Film Performance	Reference
Starch-based nanocomposite films	Corn, wheat, potato, and waxy corn starch	Natural montmorillonite (MMT)	Melt extrusion	Biodegradable; good film-forming ability	↑ Mechanical and barrier properties	[65]
	Pea and potato starch	Starch-derived nanoparticles	Solution casting	Biodegradable; good thermal stability	↑ Tensile strength; ↓ WVP and water solubility	[66]
	Sweet potato and cassava starch	Carboxymethyl cellulose nanocrystals (N-CMCs)	Solution casting	Biodegradable; nontoxic	↑ Tensile strength and elongation; ↓ WVP and moisture absorption	[67]
	Cassava starch	Cross-linked starch nanocrystals (CSNCs)	Solution casting	Biocompatible; good film-forming ability	↑ Mechanical properties; ↓ WVP	[68]
	Potato starch	MMT and TiO_2_ nanoparticles	Solution casting	Biodegradable; good thermal stability	↑ Tensile strength; ↓ WVP	[69]
	Hydroxypropyl starch	ZnO and SiO_2_ nanoparticles	Extrusion blowing	Biodegradable; good film-forming ability	↑ Tensile strength; ↓ WVP	[70]
	Active starch	Ag, ZnO, and CuO nanoparticles	Solution casting	Antimicrobial activity	↑ Tensile strength; ↓ Water solubility; ↑ Antimicrobial activity	[71]
	Ternary potato starch	MMT and TiO_2_ nanoparticles	Solution casting	Good film-forming ability	↑ Tensile strength; ↓ WVP and transparency; ↑ Opacity	[69]
Chitosan-based nanocomposite films	Chitosan	ZnO nanoparticles	Solution casting	Biodegradable; antimicrobial activity	↑ Thermal stability; inhibited foodborne pathogens	[72]
	Chitosan	ZnO nanoparticles and stearic acid	Solution casting	Biodegradable; antimicrobial activity	↑ Antibacterial activity and tensile strength; ↓ WVP	[73]
Protein-based nanocomposite films	Soy protein	Cellulose nanocrystals (CNCs) and ZnO nanoparticles	Solution casting	Biodegradable; antimicrobial activity	↑ Tensile strength; ↓ WVP and elongation; inhibited foodborne pathogens	[74]
	Whey protein	ZnO nanoparticles	Solution casting	Antibacterial activity	↑ Antibacterial activity	[75]
	Gelatin	Silver nanoparticles (AgNPs)	Solution casting	Antibacterial activity	↑ Tensile strength; ↓ Elongation; ↑ Antibacterial activity	[76]

Note: ↑ and ↓ denote increases (improvements) and decreases (reductions), respectively, in the corresponding film properties.

### 3.2. Polysaccharide-Based Nanocomposite Films

Polysaccharides are among the most important natural materials used in sustainable food packaging [77]. They are obtained from plants, algae, crustaceans, and microbial sources. The main advantages of these materials include biodegradability, renewability, low cost, film-forming ability, and safety for food-contact applications. However, most polysaccharide films absorb moisture easily because of their hydrophilic chemical structure. This reduces their mechanical strength and water vapor barrier performance, especially in high-moisture foods such as fresh meat [78].

Nanocomposite reinforcement can reduce these weaknesses (Table 2 and Table 3). The incorporation of nanofillers, including nanocellulose, nanoclay, ZnO, TiO_2_, and Ag nanoparticles, improves the structural integrity, mechanical strength, and barrier properties of polysaccharide-based films. In addition, the integration of bioactive agents, such as essential oils and their nano encapsulated forms, can increase antimicrobial and antioxidant activities [79]. These multifunctional nanocomposite films are promising for meat packaging applications because they inhibit microbial growth, delay oxidative deterioration, minimize moisture loss, and preserve the quality and shelf life of refrigerated meat products.

#### 3.2.1. Starch-Based Films

Starch is a biodegradable, renewable, affordable, and abundant polysaccharide that originates from agricultural products such as wheat, rice, corn, and potatoes. As a result of its ability to form films, good gelatinization behavior, and total biodegradation, starch is one of the most studied alternatives to synthetic polymers made from petroleum for use in food packaging [80,81]. The primary components of starch are amylose and amylopectin. The proportion and structural arrangement of each component affect how they form into films, the crystalline structure of those films, their mechanical properties and their behavior during processing. Thermoplastics based on starch may also be developed via the use of an extruder by controlling the temperature, moisture, pressure, and shear. This will increase the possibilities for starch-based biodegradable films or coatings.

However, starch-based films have several drawbacks, which limit their ability to be directly used for advanced food packaging. Starch is typically highly hydrophilic; therefore, it does not perform well as a moisture barrier, is brittle, and has limited flexibility under humid conditions. Furthermore, the incompatible nature of starch with most other polymers limits its utility in multifunctional packaging systems [82,83,84]. To overcome these limitations, various starch modification strategies have been developed. These include plasticizing starch, chemically or physically modifying starch, blending starches with polymers, and reinforcing starches with nanoparticles. For example, the addition of proteins or bioplastics (e.g., PLA, PBS, PCL) enhances both mechanical and barrier properties while retaining biodegradability [80].

**Figure 5:**
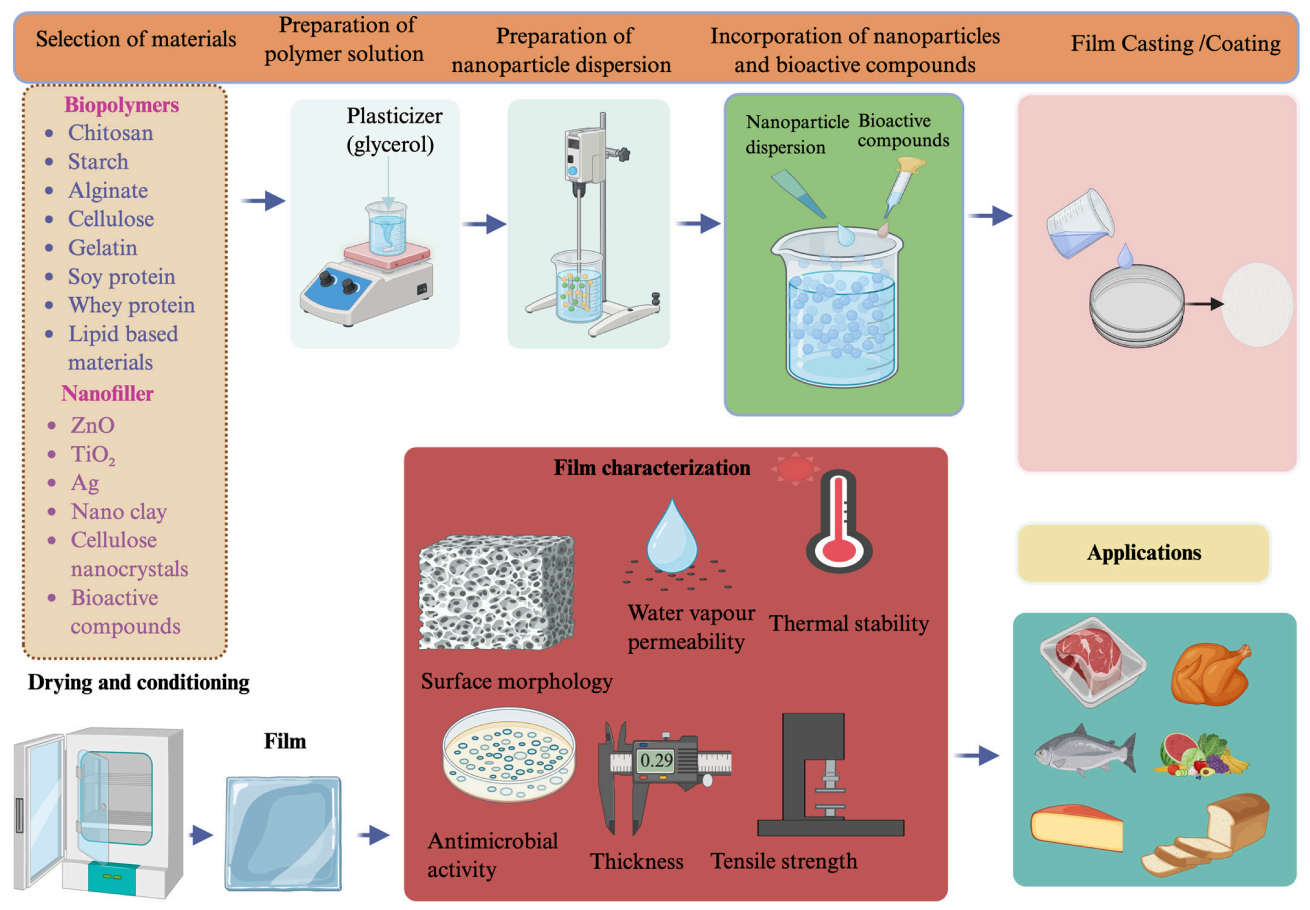
Development and application of nanocomposite films for meat packaging. The schematic illustrates the main steps involved in nanocomposite film development, including material selection, polymer solution preparation, nanoparticle dispersion, incorporation of nanoparticles and bioactive compounds, film casting/coating, drying and conditioning, and film characterization. The characterization parameters include surface morphology, water vapor permeability, thermal stability, antimicrobial activity, thickness, and tensile strength, followed by applications in meat packaging.

Among these modification strategies, nanocomposite reinforcement has attracted increasing attention because of its ability to improve the structural and functional properties of starch-based films. Nanofillers, such as starch nanocrystals, cellulose nanocrystals, and nanoclays, can strengthen the polymer matrix through improved interfacial interactions, increased crystallinity, and reduced molecular mobility. Starch nanocrystals and nanoparticles can be produced through acid or enzymatic hydrolysis, regeneration/self-assembly, and physical disintegration techniques, including high-pressure homogenization, ultrasonication, and reactive extrusion. Owing to their nanoscale dimensions and high surface area, these nanofillers can increase the tensile strength, improve the oxygen barrier properties, and reduce the water vapor permeability of starch-based films [80,85] (Figure 5). For example, de Souza Coelho et al. [86] reported that the incorporation of cellulose nanocrystals (5–15%) improved the mechanical strength and reduced the water vapor permeability of biodegradable packaging films. Similarly, Santana et al. [87] demonstrated that adding 5% maize starch nanocrystals increased the tensile strength of pea starch films by 72%, although excessive nanocrystal loading reduced film flexibility.

In addition to providing physical structure in starch-based packaging systems through nanoscale reinforcement, functional nanoparticles may impart specific active properties. These nanoparticles could include TiO_2_, ZnO and Ag, which are capable of exhibiting antimicrobial action as well as enhancing certain aspects of the mechanical and barrier properties of the polymer matrix. For example, Bagher Abiri et al. [88] demonstrated that the inclusion of nano-TiO_2_ in polyethylene/silica starch biocomposite films resulted in greater antibacterial efficacy, lower oxygen and water vapor permeability and longer shelf life of stored refrigerated chicken fillets than unmodified films did. Similar enhancements in antioxidant and antimicrobial efficacy have been observed with the inclusion of bioactive compounds, including essential oils and plant-derived extracts or their nanoencapsulated forms. However, these compounds act as functional active agents rather than nanofillers [89].

#### 3.2.2. Chitosan-Based Films

Chitosan is among the most studied marine-derived biopolymers for the development of sustainable edible antimicrobial packaging materials. Chitosan is obtained from partially deacetylated chitin that is recovered from crustaceans such as shrimp and crab shells, which provides a means to utilize seafood byproducts effectively [90]. Chitosan has been shown to have good film-forming properties, is biodegradable, biocompatible, and nontoxic and has inherent antimicrobial properties that make it ideal for use in various areas, including food packaging, pharmaceuticals, and agricultural and biomedical uses [91]. Chitosan chemically consists of linear β-(1→4)-linked polysaccharides composed of glucosamine units with varying degrees of N-acetyl glucosamine. It can be prepared from chitin via chemical alkaline deacetylation or enzymatically [92,93].

The antimicrobial activity of chitosan is associated mainly with electrostatic interactions between positively charged amino groups of chitosan and negatively charged microbial cell membranes, resulting in membrane disruption, leakage of intracellular components, and inhibition of microbial growth. In addition, chitosan can chelate essential minerals required for microbial metabolism and may interfere with intracellular processes, contributing to microbial inhibition [94,95]. When incorporated into packaging films, chitosan can provide both active antimicrobial protection and passive barrier functions by limiting microbial attachment, reducing oxygen transfer, and delaying the oxidative deterioration of food products.

Nonetheless, a major drawback of using pure chitosan films is that they are generally sensitive to moisture, show weak mechanical properties relative to those of other materials, and exhibit poor barrier characteristics. This ultimately limits them to applications with lower moisture levels, such as dried fruits and vegetables, nuts or snacks; however, there are many applications where higher moisture content is present, such as in the case of meat products [96]. The functional properties of chitosan films have been improved by modifying the films via several methods, including polymer blending, cross-linking of polymers, nanofiller incorporation into polymers, and incorporation of bioactive compounds. One method of improving the functional properties of chitosan-based packaging systems is through the use of nanocomposite reinforcement because the addition of nanoparticles can enhance the mechanical strength and barrier properties of polymeric materials through increased interfacial interactions in the material matrix [97].

ZnO nanoparticles are among the most studied nanofiller systems for chitosan films because of their ability to enhance mechanical properties and display antimicrobial effects. Souza et al. [98] demonstrated the development of chitosan-ZnO bio/nanocomposites based on ZnO nanoparticles produced via a green method from apple peel waste. Compared with untreated samples, when used as an active film in contact with fresh poultry, chitosan-ZnO bio nanocomposite films increased the time before microbial growth occurred and reduced lipid oxidation while maintaining color during the refrigeration storage period. Additionally, the increase in antioxidant capacity observed was also related to the presence of phenolic compounds associated with the biologically synthesized ZnO nanoparticles, indicating the importance of considering the synthesis methodology of the nanoparticles since it may affect the performance of the active packaging material. Similarly, Sasidharan et al. [99] reported that the addition of ZnO nanoparticles in combination with chitosan resulted in a greater shelf life extension of chicken meat, which reached 7 days, whereas the addition of ZnO produced through chemical methods yielded an additional shelf life extension of two days with respect to a pure chitosan film. These results indicate that the use of films reinforced with ZnO nanoparticles could be considered a strategy for extending shelf life and improving the quality retention of meat products.

Recent studies have examined the use of combinations of chitosan with other biodegradable polymers and plant-derived antimicrobials as a means of enhancing preservative action through synergy. A recent study by Pires et al. [100] developed chitosan/nanocellulose-based films containing sage essential oils for use in the packaging of fresh poultry meat. Nanocellulose was used to improve the physical properties of the films and reduce the migration of the essential oils, whereas the presence of the bioactive components increased the antimicrobial and antioxidant activities. Films made from this system were shown to be capable of reducing the microbial load of samples by 2–3 logs and decreasing lipid oxidation compared with those of control samples. However, one of the major challenges associated with films that are designed to contain high levels of volatiles is the ability to maintain control over their release; in many cases, strongly volatile chemicals can negatively impact food flavor and, ultimately, consumer acceptability. Similar results have been reported for films developed from chitosan/pullulan blends, which demonstrate considerable promise for application in active meat packaging systems. In addition to providing an excellent transparent film-forming agent, pullulan adds significant antimicrobial functionality to films because of its interaction with chitosan [101].

Moreover, incorporating natural antimicrobial compounds into chitosan-based films further enhances their preservation performance. For example, Xiao et al. [102], reported that chitosan/pullulan and chitosan/pullulan/carvacrol films were able to inhibit both bacterial development and TVB-N generation in refrigerated goat meat. They also determined that the addition of carvacrol to the film inhibited the development of *Pseudomonas* and extended the shelf life of the product to 13 days. To reach an optimal level of efficacy while not degrading the sensory characteristics of the final product, optimizing the concentrations of bioactive compounds is essential. Owing to their ability to provide controlled release of active compounds over time, recent advancements in controlled-release technologies, including nanoemulsion-based packaging and multilayered films, are attracting interest. Mutlu and Mor [103] developed multilayered chitosan films that contained rosemary oil nanoemulsions. These compounds demonstrated increased antimicrobial action against *Staphylococcus aureus* and *Bacillus subtilis*. Additionally, during minced beef storage at 4 °C, these films decreased the presence of *Pseudomonas* spp. and H2S-producing bacteria while retaining acceptable oxidative stability criteria. Collectively, these findings demonstrate that advanced chitosan-based systems can be used for the controlled delivery of natural antimicrobial agents and prolonged preservation of meat products. The combination of biodegradability, active antimicrobial functionality, enhanced mechanical performance, and controlled-release capability highlights the strong potential of these materials as next-generation sustainable packaging materials for meat preservation.

#### 3.2.3. Cellulose-Based Films

The use of cellulose and its derived products are the most studied renewable biopolymer options for sustainable meat packaging. The main reasons are that they can be easily biodegradable, form films well, and provide a good barrier against oxygen [104,105]. Native cellulose has poor solubility and is poorly processed; thus, it cannot be directly used as an option for packaging. This is why there has been increased interest in cellulose derivatives, which include carboxymethyl cellulose (CMC), methyl cellulose, hydroxypropyl methylcellulose (HPMC) and bacterial cellulose. They have better processability and function than native cellulose does [106]. Bacterial cellulose provides superior mechanical strength and structure because it has a highly connected nanofibrillar network [107]. CMC provides a lower cost, easier processing, and compatibility with other biopolymers [108].

Despite these advantages, the widespread application of cellulose-based films in meat packaging is constrained by their hydrophilic nature. The abundance of hydroxyl groups promotes water absorption, leading to high water vapor permeability (WVP), dimensional instability, and deterioration of mechanical properties under the high-humidity conditions associated with fresh meat storage [109]. Consequently, cellulose films alone rarely provide adequate moisture protection for commercial meat packaging.

To address these limitations, nanoparticles such as ZnO, TiO_2_, silica nanoparticles, nanoclays, and cellulose nanocrystals (CNCs) are incorporated into cellulose matrices [110]. Rather than simply acting as fillers, these nanoparticles function as impermeable domains that force water molecules to follow a more tortuous diffusion pathway, thereby reducing WVP [111]. Simultaneously, they act as physical cross-linking sites between cellulose chains, generating a denser polymer network with improved mechanical strength and a reduced free volume for moisture diffusion. Similarly, hydrophobic essential oils dispersed as microdroplets decrease the affinity of the polymer matrix for water while providing antimicrobial and antioxidant functions [11]. However, these improvements are not unlimited. Excessive nanoparticle loading frequently causes agglomeration, creating structural defects that reduce barrier efficiency and may even impair mechanical performance [112]. Therefore, the performance of cellulose nanocomposites depends more on nanoparticle dispersion and interfacial compatibility than on nanoparticle concentration alone.

Optical performance presents another important requirement for cellulose-based packaging films. These packaging films need to possess sufficient transparency within the visible spectrum so that consumers are able to evaluate their contents and provide adequate protection from ultraviolet (UV) irradiance [113]. UV exposure accelerates photooxidative reactions of the lipid, protein, pigment, and vitamin components of meat products; such reactions result in rancid flavors, loss of color, and loss of nutrients and ultimately shorten the overall shelf life of the meat product. Consequently, UV blocking ability is a significant function in maintaining the quality of the meat product. The utilization of zinc oxide (ZnO) nanoparticles in hydroxyethyl-cellulose composites offers an efficient method for enhancing the UV barrier functionality of cellulose films. This is due to the large bandgap semiconducting properties of ZnO particles, which absorb high-energy UV photons via electron transition from the valence band to the conduction band and thus reduce UV transmission through the packaging material. The hydroxyethyl-cellulose/ZnO composite films exhibited improved UV absorption characteristics and effectively blocked the UV radiation that was responsible for oxidative deterioration. However, excessive addition of ZnO into these composite films results in decreased visible transparency since higher concentrations of particulates and aggregation of particulates increase light scattering, resulting in undesirable coloration. Thus, the ZnO concentration, particle size and dispersal characteristics must be optimized to obtain optimal transparent visibility for consumers and UV protective functions [11].

Recent studies have shown that cellulose nanocomposites with multiple functions may be better at preserving than conventional cellulose-based films for use as packaging materials for fresh meats. A bacterial cellulose/polypyrrole/ZnO nanocomposite package was developed by Pirsa and Shamusi [114] that had antimicrobial properties against both *psychrotrophic* and *mesophilic* microorganisms and stabilized the pH of refrigerated chicken meat. Films made from carboxymethylcellulose (CMC), although inexpensive and easily processible, do not exhibit the same level of mechanical strength as those made from bacterial cellulose; however, they do offer improved barrier properties because of their greater surface area and stronger interaction with the polymer matrix [115]. As such, overall, cellulose-based nanocomposite packaging appears to offer a promising alternative to petroleum-based packaging materials; however, several limitations need to be overcome. Cellulose-based packaging is still sensitive to moisture, large-scale manufacturing of bacterial cellulose is expensive, and achieving a uniform distribution of nanoparticles during industrial-scale manufacturing processes is still an issue. Therefore, future research should focus on modifying the surface of nanocellulose, creating multilayer composite packaging, developing controlled release antimicrobial technologies and developing cost-effective methods for mass-producing cellulose-based packaging wraps for meat products.

#### 3.2.4. Alginate-Based Films

Alginate is a type of anionic, linear polysaccharide that is extracted primarily from brown algae. It has both β-D-mannuronic acid (M) and α-l-guluronic acid (G) residues, but there are differences in the M/G ratios of the specific alga used to extract it [116]. The alginate extracted from Laminaria hyperborea contained approximately 60% G units. However, most commercial alginate extracted from other types of algae usually has much lower G contents in their fractions (14–31%) [117]. The G unit location/abundance also impacts how gels form when G-rich areas interact with divalent cations such as Ca^2+^, forming three-dimensional ionic networks [118]. Owing to these physical properties, in addition to being easily broken down and nontoxic to humans or animals and forming good films, alginate has been used as a base material for creating food coatings and packaging films.

Additionally, alginate films have inherent constraints, limiting their use directly as meat packaging. Alginate has many hydroxyl and carboxyl groups, which makes it extremely hydrophilic; this results in very high WVP values and poor moisture barrier properties. Furthermore, the weak molecular forces within the polymeric matrix result in low mechanical strength. Under high RH, the mechanical strength can be significantly lower than that at ambient temperature and RH. As such, when considering the protection of moisture-sensitive foods, alginate generally cannot protect the product sufficiently enough by itself; therefore, it will need either structural modifications or incorporation into films that contain additional functional materials. Antimicrobial applications using alginate generally do not exhibit significant intrinsic antimicrobial activity and are primarily utilized as a film matrix material for the inclusion of active agents, i.e., essential oils, plant extracts, bacteriophages, antioxidants and [119]. Therefore, the antimicrobial efficacy of films containing alginate is dependent upon the type, stability and release characteristics of the included bioactive agent. Similarly, Lourenço et al. [120] incorporated encapsulated pineapple peel extracts into alginate films and reported reduced *Pseudomonas* spp. growth, delayed lipid oxidation, and improved color stability of beef steaks during refrigerated storage. Similarly, calcium-crosslinked sodium alginate films containing the bacteriophage ϕIBB-PF7A reduced *Pseudomonas fluorescens* populations on chicken breast [121].

More recently, Yang et al. [122] developed a film containing oregano essential oil/β-cyclodextrin inclusion complexes in an alginate coating that achieved 71.6% encapsulation efficiency; this improved the stability of the essential oil by means of controlled release. The system also reduced the pH, TBARS value, TVB-N formation and microbial activity during storage. Several modification techniques for overcoming the limitations associated with alginate films are being studied. These include crosslinking, polymer blending, incorporating hydrophobic materials, and producing nanocomposites. By creating ionic junctions between the chains of alginate via cross-linking with calcium ions, it is possible to increase the density of the network structure of the film, reduce its capacity for movement and restrict water permeability. In addition, the addition of hydrophobic material may be used to increase the resistance of the film to moisture. For example, the addition of avocado leaf extracts decreased the WVP of alginate films from 21.53 to 11.21 g mm/m^2^ day kPa. This was accomplished by limiting water interactions with hydrophilic functional groups [123].

The use of nanofillers to enhance alginate film characteristics is an additional method to obtain better performing films. When incorporated into a polymer matrix at the nanoscale, particles (e.g., ZnO and sulfur) can be used as barriers that are impermeable to water vapor. The presence of these particles in a polymer matrix generates tortuous pathways that increase the path length for diffusing molecules and thus limit the rate of water vapor passage. In addition, when evenly dispersed, nanoparticles can also physically interact with alginate molecular chains, thereby acting as physical cross-links. This led to the formation of a more compact network structure and therefore increased both the barrier function and the mechanical strength of the alginate film [123,124]. However, the extent to which nanoparticles function effectively to improve barrier function depends heavily upon the amount of nanoparticles present, how they are dispersed in the alginate film matrix and whether they have sufficient compatibility with the matrix. Overloading an alginate film matrix with too many nanoparticles could disrupt the interactions between alginate molecular chains, generate defects in the polymer film structure and result in increased water vapor permeability (WVP) rather than improved barrier function [125,126].

### 3.3. Protein-Based Nanocomposite Films

Proteins are attractive raw materials for food packaging films because of their excellent film-forming ability and structural versatility. The presence of diverse amino acid residues provides proteins with various functional groups (e.g., amino, carboxyl, hydroxyl, and sulfhydryl groups), enabling hydrogen bonding, electrostatic interactions, hydrophobic interactions, and covalent crosslinking with other polymers or active compounds. These molecular interactions allow protein-based films to be tailored for improved mechanical, barrier, and functional properties [127,128]. The characteristics of protein films strongly depend upon the source of the proteins used and the conditions under which the experiment was carried out. Protein films exhibit high strength and viscoelastic properties; nevertheless, these desirable properties of protein films are shadowed by two primary drawbacks: brittleness and high sensitivity to moisture [129]. Furthermore, the utilization of proteins in food packaging materials is limited by the low mechanical strength, brittleness, poor barrier, and physical and chemical characteristics of the material. To overcome this limitation, many studies have been carried out recently using nanomaterials in biobased polymers known as bionanocomposites. The incorporation of nanomaterials in biopolymers will improve not only the barrier properties but also the thermal stability and mechanical properties without affecting biodegradability [129,130,131]. Whey protein isolates, soy protein isolates, and gelatin are among the commonly used proteins in bionanocomposites.

#### 3.3.1. Gelatin-Based Films

Gelatin appears to be an attractive option as a protein-based alternative to traditional materials for packaging meat because its properties allow it to form transparent, elastic films with high barrier properties against oxygen at relatively low-to-moderate levels of relative humidity. Gelatin is generally produced via the use of collagen-rich raw materials, including skin and bones, thus supporting the concept of waste reuse. Amjadi et al. [132] developed a gelatin-chitosan nanofiber-ZnO nanoparticle composite film that contained betanin nanoliposomes for use on fresh beef. This film exhibited a water contact angle of 92.49°. A contact angle of this magnitude indicates increased hydrophobicity of the surface of the film compared with that of nontreated films, and a DPPH radical-scavenging activity of 53.02% was observed. The gelatin composite films inhibited the growth of bacteria introduced into beef during storage, limited lipid oxidation, reduced the pH, and limited color degradation. Similarly, Sayadi et al. [133] examined 24-day storage of chickens at 4 °C in gelatin films containing 1% TiO_2_ nanoparticles, 2% cumin essential oil, or a combination of both. The pure gelatin and nanocomposite films decreased bacterial proliferation by approximately three logarithmic cycles over the course of 24 days. However, the gelatin + TiO_2_ + cumin essential oil composite film demonstrated the most significant antimicrobial effects. Likewise, *Listeria monocytogenes* grew less effectively throughout storage. In addition to having lower TVB-N values than untreated control chickens did, the treated chickens presented reduced lipid oxidation values and better sensory attributes for the period between four and twelve days. Importantly, it appears that cumin essential oils provided a greater contribution toward improved oxidative stability; conversely, TiO_2_ demonstrated greater contributions to enhancing antimicrobial efficacy as well as overall barrier effectiveness. Thus, these studies indicate that the application of a single type of nanoparticle will likely not result in adequate preservation on its own; rather, incorporating nanofillers along with naturally occurring antioxidants could be an important method to reduce microbial growth and oxidative degradation simultaneously.

More recent research has shown that gelatin systems can also be designed for controlled release. Chu et al. [134] developed a gelatin-highland barley β-glucan film containing MCM-41 mesoporous silica nanoparticles loaded with cinnamaldehyde and carvacrol. The film released 42.8% of the active compounds over 30 days at 4 °C but 73.6% at 25 °C, indicating temperature-responsive release. When a ready-to-eat steak was applied, the shelf life of the film doubled to 18 days at 4 °C, and the shelf life increased to 12 days at 25 °C. This is a stronger design than simple additive films because the release rate responds to the storage temperature and microbial growth pressure. It is especially relevant for ready-to-eat meat, where consumers may not cook the product again before eating. However, the evidence also shows trade-offs. Similarly, Sultan et al. [135] studied gelatin films containing cinnamaldehyde and ZnO nanoparticles. ZnO reduced the water vapor permeability by 12.07% and the oxygen permeability by 86.86%**,** reduced the monolayer moisture content by 35.79%, and reduced the film solubility by 15.15%. These are strong barrier improvements. However, ZnO also reduced the tensile strength by 13.32%**,** reduced the Young’s modulus by 18.33%, and lowered the antioxidant activity by 9.09%. This shows a key limitation: the addition of nanoparticles may improve the barrier and antimicrobial properties but weaken the mechanical strength and antioxidant performance.

Overall, gelatin-based nanocomposite films can extend the shelf life of meat, reduce microbial growth, delay lipid oxidation, and improve barrier properties. However, gelatin is most effective when it is used in hybrid systems with chitosan, ZnO, TiO_2_, essential oils, β-glucan, nanoliposomes, or mesoporous carriers. For meat packaging, gelatin films should therefore be designed as product-specific systems, beef needs oxidation and color control, poultry needs strong antimicrobial protection, seafood needs rapid spoilage control, and ready-to-eat meat benefits from controlled-release antimicrobial systems. The best gelatin-based film is not the one with the highest nanoparticle level but the one that provides balanced preservation, acceptable sensory quality, safe migration behavior, and stable mechanical performance [136,137].

#### 3.3.2. Soy Protein-Based Films

Soy protein is one of the most researched plant proteins that can be used in biodegradable food packages because it is abundant, renewable, relatively inexpensive and has good film-forming properties. Among commercial soy protein products, soy protein isolate (SPI) is the most commonly used because of its high protein content (>90%) and the presence of functional groups from protein fractions such as β-conglycinin and glycinin. These reactive groups facilitate interactions with plasticizers, cross-linking agents, nanofillers, and bioactive compounds, allowing modification of the film structure and functionality [138]. However, SPI films are inherently weak in terms of water vapor permeability and mechanical integrity and can be used as packaging materials for high-water-content foods. Consequently, recent research has focused on the development of nanoreinforced SPI films, as well as those containing functional or “active” components. For example, Xiao et al. [74] prepared SPI films using cellulose nanocrystals (CNCs), ZnO nanoparticles, and CNC/ZnO nanohybrids. The incorporation of CNCs into the film matrix resulted in improvements in the mechanical strength, gas barrier properties, and thermal stability relative to those of pure SPI films. In addition, ZnO exhibited antibacterial properties against *E. coli* and *S. aureus*. Importantly, the CNC/ZnO hybrid was found to significantly reduce Zn migration from the film matrix. Likewise, the application of SPI combined with both nanoclays and essential oils as an active package has been evaluated. Khosravi et al. [139] demonstrated that SPI montmorillonite composite films containing nano emulsified Zataria multiflora essential oil inhibited microbial growth and lipid oxidation and preserved the shelf life of chilled chicken burgers stored under refrigeration conditions.

SPI films have also been formulated as part of smart packaging systems. Antioxidant-active films containing grape-skin anthocyanin loaded into SPI/ZnO films were produced by Ran et al. [140], which displayed both pH-sensitive color changes and promising results when these types of films were used to monitor pork freshness in real time. The water solubility of these films is still an issue that needs to be addressed before they can be used practically for food packaging. There are several other ways in which ZnO can improve SPI film performance, including its antimicrobial and UV blocking properties, as well as improve the mechanical strength of SPI films and their barrier function [141,142]. High levels of nanoparticles may add to the cost of film production and create concern over whether there will be acceptable migration of the nanoparticles from the film into food products.

#### 3.3.3. Whey Protein-Based Films

Whey protein is a biodegradable material that is utilized in food packaging because it has the ability to form films, is transparent and biodegradable, and has barrier properties with respect to oxygen, volatile compounds and oils [143]. Whey protein isolate (WPI) is one of the most important byproducts of cheese and casein production; therefore, WPIs can form cohesive protein networks that are very useful for making edible films and coatings. To increase the flexibility of whey protein films, glycerol is generally employed as a plasticizer [144]. Furthermore, the hydrophilicity of whey protein results in high water vapor permeability and limited mechanical resistance, which restricts its use in products sensitive to moisture [145].

However, nanocomposite reinforcement and the incorporation of bioactive compounds have been investigated to improve the mechanical, barrier, and active properties of WPI-based packaging systems. The incorporation of nanofillers and bioactive agents has transformed whey protein films from passive barriers into multifunctional packaging materials. Cellulose nanofibers and polydextrose have been reported to improve the mechanical properties and reduce the water vapor permeability of whey protein films [146]. Similarly, Hajjar Bargh et al. [147] developed WPI coatings containing *Ziziphora clinopodioides* essential oil for refrigerated chicken fillets. The optimized coating containing 1% essential oil showed improved antimicrobial activity and reduced spoilage indicators, including microbial growth and TVB-N formation. Similarly, Sheerzad et al. [148] demonstrated that a hybrid WPI coating incorporating nanochitosan, bacterial nanocellulose, and cinnamon essential oil synergistically inhibited microbial proliferation, delayed lipid oxidation, and extended the refrigerated shelf life of chicken fillets by more than five days compared with untreated controls. These findings demonstrate that the synergistic combination of whey proteins, nanostructured reinforcements, and bioactive compounds substantially enhances the structural integrity and preservation performance of WPI-based packaging systems. Consequently, whey protein nanocomposites show considerable potential as sustainable active packaging materials for both poultry and red meat preservation [149].

### 3.4. Lipid-Based Nanocomposite Films

Lipid-based films and coatings have been shown to be good at reducing the moisture content in a product, which improves its shelf life, because they have good hydrophobic properties [150]. The barrier characteristics of lipid films depend on the structural organization of the film matrix; the physical properties of the lipid molecules, such as their crystalline structure, molecular weight of fatty acids, number of double bonds in the acyl chains and functional groups present on the molecules [151]. The rate of passage through a lipid film is also affected by interactions with molecules that can pass through it and how the lipid film is formed. Lipids (such as waxes) tend to inhibit water vapor transfer because they are nonpolar; therefore, these types of lipids are often included in biodegradable packaging.

However, pure lipid films generally exhibit poor mechanical properties, brittleness, greasiness, opacity, and limited adhesion to moist food surfaces, which restricts their application as standalone packaging materials [152]. Therefore, lipids are commonly integrated with hydrophilic biopolymers, including starch, cellulose, chitosan, pectin, alginate, carrageenan, gelatin, and protein-based materials, to form emulsion films, bilayer films, or multilayer coatings. In these composite systems, the hydrophilic polymer phase provides structural integrity, film-forming ability, and mechanical strength, whereas the lipid phase improves moisture resistance by reducing water vapor diffusion. Moreover, intermolecular interactions and physical cross-linking between lipid and polymer components can increase the tensile strength and thermal stability while maintaining favorable barrier properties [151]. Several studies have demonstrated the effectiveness of lipid incorporation in improving the performance of biodegradable films. Shen et al. [153] reported that oleic acid incorporation into wheat bran cellulose/wheat gluten composite films significantly decreased water vapor permeability and water solubility due to increased hydrophobicity. Similarly, da Silva Pereira et al. [154] reported that palmitic and caproic acids improved the water resistance of fish protein films. Waxes are also among the most effective lipid components for moisture protection because their highly hydrophobic crystalline structures create strong resistance against water vapor migration [155]. These findings indicate that lipid incorporation is an effective strategy to overcome the inherent moisture sensitivity of polysaccharide- and protein-based films.

Recent advances in nanotechnology have further expanded the functionality of lipid-based films by enabling the development of lipid nanocarriers and lipid–nanofiller hybrid systems. In meat packaging, these systems are particularly valuable because meat deterioration is strongly associated with moisture loss, microbial growth, lipid oxidation, and discoloration. Lipid components can limit water transfer, whereas nanoparticles or encapsulated bioactive compounds can provide antimicrobial and antioxidant activity, producing multifunctional packaging materials rather than simple moisture barriers. For example, Ghasemi et al. [43] developed an edible nanocomposite coating for beef based on carboxymethyl cellulose, oleaster mucilage, ZnO nanoparticles, and nanostructured lipid carriers (NLCs) containing savory essential oils. The optimized NLC system showed 71.4% essential oil loading and >98.7% encapsulation efficiency. A coating containing 3.4% NLCs generated a 17 mm inhibition zone and extended beef preservation for 12 days by reducing microbial growth (>1.6 log CFU/g reduction), lipid oxidation (lower thiobarbituric acid values), pH changes, and total volatile nitrogen formation. These results highlight the potential of lipid nanocarriers to protect volatile antimicrobial compounds, regulate their release, and increase their effectiveness during storage. Similarly, Pandey et al. [156] developed a chitosan/alginate bilayer cling film containing a lavender-ginger essential oil nanoemulsion combined with silver nanoparticles. The resulting film exhibited a thickness of 22.5 μm, improved mechanical strength and thermal stability, maintained a red meat color for up to 7 days, and reduced microbial growth. This study demonstrated that lipid-derived active compounds achieve greater preservation efficiency when stabilized within a robust biopolymer matrix than when applied directly. Overall, lipid-based nanocomposite films provide an effective approach for developing active meat-packaging materials by integrating moisture resistance with antioxidant and antimicrobial functions. However, their practical application remains limited by challenges related to lipid dispersion, mechanical weakness, transparency, sensory acceptance, nanoparticle migration, cost, and industrial-scale processing [37]. Future research should therefore move beyond laboratory-scale antimicrobial evaluation and emphasize controlled-release mechanisms, long-term storage performance, food-contact safety assessment, regulatory approval, life-cycle impacts, and scalable manufacturing strategies. Such considerations are essential for translating lipid-based nanocomposite packaging from experimental systems into commercially viable sustainable food-packaging solutions.

## 4. Application of Nanocomposite Films and Coatings in Meat Products

### 4.1. Fresh Red Meat

Red meat represents a rich source of nutrients such as vitamins, minerals, trace elements, fatty acids and high-quality proteins. However, red meat has a higher moisture level and is richer in nutrients than many other foods are and therefore is extremely vulnerable to the effects of lipid oxidation, microbial spoilage, protein degradation and enzymatic breakdown. The browning that occurs due to the oxidation of myoglobin as well as several chemical reactions may impact consumers’ perceptions of freshness and quality since discolored meat is typically perceived as less fresh or less desirable [43]. Therefore, effective preservation strategies are needed to extend shelf life while maintaining nutritional value, sensory characteristics, and safety.

Conventional meat preservation techniques, including salting, drying, smoking, fermentation, refrigeration, and freezing, extend shelf life by reducing the water availability for microorganisms or slowing their metabolic activity. However, these approaches often have limitations, including effects on sensory and nutritional quality, high energy consumption and a limited ability to meet consumer preferences. As a result, interest in the use of new preservation technologies, including high-pressure processing, irradiation, modified atmosphere packaging and edible coatings to improve the long-term shelf life of fresh meats, has increased [157]. Active biodegradable films and coatings are emerging as good alternatives for existing products that include barrier properties in addition to antimicrobial and antioxidant functionalities. Polysaccharides based on CMC, sodium alginate, starch and chitosan are being studied extensively because of their degradability, ability to form films and compatibility with bioactive substances [158]. However, some biopolymers, such as CMC, lack intrinsic antimicrobial activity; therefore, the incorporation of antimicrobial agents, including essential oils, plant extracts, or nanoparticles, is commonly applied to increase their functionality.

A number of nanofiller materials are being researched for food packaging due to several advantages, including antimicrobial properties, lower toxicity than some other fillers and increased physical barrier properties. For example, films produced from a combination of CMC (carboxymethyl cellulose), okra mucilage and ninc oxide NPs were shown to reduce bacterial loads on the surface of chicken products while improving the storage life of the product under refrigeration [46]. Similarly, chitosan/PVA/gelatin films that included ZnO NPs also improved the film properties, which resulted in a longer shelf life for meat when used as active biodegradable nanocomposite packaging systems [159]. These findings highlight the potential of active biodegradable nanocomposite packaging systems for maintaining fresh meat quality.

**Table 3:** Applications of nanocomposite films and coatings in meat and meat product preservation.

Film Category	Biopolymer Matrix	Nanomaterial/Active Agent	Main Film Properties	Application	Reference
Starch-based	Potato starch/apple peel pectin	ZrO_2_ nanoparticles	Reduced water vapor permeability; improved antibacterial and mechanical properties	Quail meat	[160]
	Potato starch/watermelon peel pectin	Nano-TiO_2_	Improved mechanical, antioxidant, and antibacterial properties	Tan mutton	[161]
	Sago starch	*Peganum harmala* extract and TiO_2_	Improved mechanical and barrier properties; enhanced oxidative stability	Chicken fillets	[162]
	Tapioca starch	Cellulose nanocrystals	Increased tensile strength; reduced elongation, water vapor permeability, and *L. monocytogenes* growth	Ready-to-eat chicken meat	[163]
	Pullulan/cassava starch	Caseinate/zein nanoparticles loaded with LC-EO	Reduced bacterial growth, color change, pH change, texture loss, and lipid oxidation	Beef meat	[164]
	Tamarind seed starch	FeO and ZnO nanoparticles	High oxygen-scavenging and antimicrobial activity	Chicken meat	[165]
	Potato starch	TiO_2_ nanoparticles	Improved barrier and mechanical properties; reduced oxidation and spoilage bacteria	Chilled meat	[166]
Chitosan-based	Chitosan	Nisin-pectin nanoparticles loaded with anthocyanins	Improved barrier and mechanical properties	Tilapia fish meat	[167]
	Chitosan/PVA/gelatin	ZnO nanoparticles	Improved barrier, mechanical, thermal, and antimicrobial properties	Chilled chicken meat	[168]
	Chitosan/ginger essential oil	MMT	Improved mechanical and barrier properties; delayed fat oxidation and microbial growth	Chilled beef	[169]
	Chitosan	ZnO nanoparticles	Reduced oxidation and microbial growth; improved antioxidant activity	Fresh poultry meat	[98]
	Chitosan/*Cymbopogon citratus* essential oil	TiO_2_ nanoparticles	Improved mechanical properties; extended shelf life by reducing bacterial growth	Minced meat	[170]
	Chitosan/pullulan	Carvacrol/thymol nanoemulsions	Improved mechanical, barrier, antioxidant, and antibacterial properties	Pork	[171]
Cellulose-based	Gelatin/ethyl cellulose	Silver nanoparticles	Improved barrier and mechanical properties	Sliced meat	[172]
	Whey protein isolate/cellulose	TiO_2_	Improved antimicrobial properties	Lamb meat	[173]
	Carboxymethyl cellulose	ZnO nanoparticles	Improved antimicrobial activity and oxidative stability	Chicken breast meat	[46]
	Carboxymethyl cellulose	Nanomontmorillonite	Improved antimicrobial and antioxidant properties	Minced camel meat	[174]
Alginate-based	Sodium alginate/cinnamaldehyde	Ag/ZnO nanoparticles	Strong antibacterial activity against multidrug-resistant pathogens	Chicken meat	[175]
	Chitosan/alginate	Silver nanoparticles	Improved mechanical strength, thermal stability, and antimicrobial activity against *E. coli*	Red meat	[156]
	Sodium alginate	CuO nanoparticles	Improved antimicrobial activity	Chicken meat	[176]
	Alginate/cumin essential oil	TiO_2_	Improved antimicrobial activity and reduced oxidation	Fresh beef	[133]
Protein-based	Gelatin/chitosan	ZnO nanoparticles	Improved antioxidant, antimicrobial, and mechanical properties	Fresh beef	[132]
	Gelatin	TiO_2_	Improved antimicrobial activity and reduced lipid oxidation	Fresh chicken meat	[133]
	Gelatin/carrageenan	Zein nanoparticles	Improved mechanical and biological properties	Chicken meat	[177]
	Whey protein isolate/cellulose	TiO_2_ nanoparticles	Improved antimicrobial and organoleptic properties	Lamb meat	[173]
	Whey protein	ZnO nanoparticles	Improved antioxidant and antimicrobial properties	Fish meat	[178]
	WPI/cellulose nanofiber	TiO_2_ nanoparticles	Improved antibacterial properties	Lamb meat	[173]
	Soy protein isolate	TiO_2_	Strong antimicrobial activity	Carp fillet	[178]
	Soy protein isolate	ZnO nanoparticles	Improved barrier properties, tensile strength, and thermal stability	Pork	[74]
	Soy protein isolate	Montmorillonite nanoclay	Improved antimicrobial properties	Chilled chicken burgers	[139]
	Soy protein isolate	ZnO nanoparticles	Improved crystallinity and thermal stability	Pork	[140]

### 4.2. Poultry and Processed Meat Products

Appropriate packaging is important for maintaining both the safety and quality of a product and extending the shelf life of a perishable food, such as poultry or processed meat. As these types of foods are particularly susceptible to spoilage due to a variety of factors, including microbial contamination, lipid degradation (oxidation), loss of moisture and color changes, the development of novel packaging technologies that utilize advanced nanomaterials has recently emerged as a viable solution. The application of nanomaterials in composite film structures provides significant improvements in reducing microbial activity, slowing oxidation processes and preserving the quality of meats during refrigerated storage [179].

Nanoencapsulation of essential oils represents an effective strategy for overcoming the volatility, poor stability, and rapid release associated with free essential oils while improving the functional performance of biodegradable films. For example, incorporating nanoliposomal garlic essential oil into chitosan films enhanced film flexibility, water resistance, antioxidant capacity, and antimicrobial activity, resulting in reduced pH, TVB-N, peroxide value (PV), TBARS, and microbial growth in refrigerated chicken breast fillets [180]. However, increasing the concentration of nanoliposomal garlic essential oil reduced the tensile strength, indicating a trade-off between functional activity and mechanical performance. Notably, although 2% garlic essential oil had the strongest antimicrobial effect, a concentration of 1% achieved comparable preservation efficiency with less active ingredients, highlighting the importance of optimizing active agent loading to balance preservation efficacy, film properties, and cost-effectiveness. Similarly, Sasidharan et al. [99] investigated chitosan nanocomposite films containing biogenic and chemically synthesized ZnO nanoparticles for poultry meat packaging. Compared with the chitosan, PVA, and unwrapped samples, both films reduced microbial growth, lipid oxidation, weight loss, pH changes, and color deterioration. The biogenic ZnO nanocomposite performed better overall, extending the acceptable shelf life to seven days, compared with five days for the chemogenic ZnO film and four days for the chitosan film. Among the tested formulations, 0.5% biogenic ZnO provided the best control of microbial growth and lipid oxidation. However, zinc migration into the meat increased, especially at higher nanoparticle concentrations, indicating the need for further assessment of its cytotoxicity, consumer exposure, and long-term safety.

Youssef et al. [181] further developed a bionanocomposite coating based on chitosan, polyvinyl alcohol, TiO_2_ nanoparticles, and cinnamon essential-oil nanoemulsions for refrigerated chicken breast fillets. Increasing the nanoemulsion concentration from 5% to 20% increased the antioxidant and antimicrobial activities, reduced water-vapor transmission, and limited moisture-related weight loss. The coatings inhibited aerobic and anaerobic bacteria, coliforms, psychrotrophic microorganisms, *Staphylococcus aureus*, *Listeria*, yeasts, and molds. The formulation containing the 20% cinnamon nanoemulsion produced the strongest preservation effect and maintained acceptable bacterial counts for more than 14 days. This study demonstrated that combining metal-oxide nanoparticles with nanoemulsified natural compounds can produce synergistic antimicrobial and antioxidant effects in poultry packaging. Collectively, these studies show that nanoencapsulated essential oils and metal-oxide nanoparticles can improve microbial control, oxidative stability, moisture retention, and refrigerated shelf life of poultry products.

### 4.3. Seafood and Ready-to-Eat Meat Products

Active packaging films and edible coatings are increasingly used as an active packaging strategy for both fresh and ready-to-eat (RTE) meats and fish to control microbial growth; slow the oxidative reactions that can occur during storage; monitor freshness through changes in color, texture and odor; or prolong shelf life. Essential oils have shown promising results as natural antimicrobial agents for preventing the presence of major pathogens, such as *Staphylococcus aureus*, *Pseudomonas aeruginosa* and *Escherichia coli*, when incorporated into edible coatings for use with seafood [182].

Chitosan-based packaging has been shown to be a viable option for preserving meat through its unique film-forming properties, degradable nature, and inherent antimicrobial activity [183,184]. A composite of polylactic acid and chitosan was used to form a film that was able to inhibit microbe growth on beef when it was refrigerated at 4 °C [185]. The use of chitosan films also inhibited both *E. coli* and *S. typhimurium* bacteria in chicken mince but had greater antibacterial efficacy against *E. coli* [186]. Additionally, Li et al. [187] demonstrated that heat treatment combined with a chitosan coating suppressed bacterial growth while preserving the volatile flavor compounds associated with braized duck meat after refrigeration.

The incorporation of bioactive plant extracts into chitosan-based films further enhances seafood preservation by providing synergistic antioxidant and antimicrobial effects. Phenolic-rich extracts, such as cinnamon and tea, effectively inhibit lipid oxidation and improve the storage stability of free radical-scavenging and oxidative chain-braking mechanisms in fish fillets [188]. Similarly, the incorporation of zinc oxide nanoparticles (ZnO-NPs) enhances antimicrobial activity while reducing protein and lipid oxidation during storage, thereby preserving seafood quality [189]. In addition to their bioactive functions, nanofillers also reinforce biodegradable polymer matrices by improving their mechanical strength and reducing their oxygen and moisture permeability. However, the preservation performance of these nanocomposite systems depends on the type and concentration of bioactive agent, nanoparticle dispersion, and compatibility with the polymer matrix. Therefore, optimizing the formulation parameters is essential to achieve an appropriate balance between preservation efficacy, film functionality, and food safety. In addition to preservation, chitosan-based materials have been developed for use in intelligent packaging systems. Alizadeh-Sani et al. [190] developed a pH-responsive film based on chitosan and methylcellulose with berry anthocyanins for real-time monitoring of lamb meat freshness. This film showed color changes due to pH variations and volatile basic compound accumulation during meat spoilage; both effects provided rapid indications of quality degradation. Overall, these studies demonstrated that chitosan, essential oils, plant extracts, metal oxide nanoparticles, and anthocyanins can be incorporated into active and intelligent nanocomposite packaging systems to increase microbial safety, antioxidative stability, freshness monitoring, shelf life extension of seafood and ready-to-eat meats.

## 5. Safety, Toxicity, and Regulatory Considerations

The use of nanocomposite-based technologies for meat packaging offers a number of ways to enhance the functional capabilities of packaging material through enhanced antimicrobial efficacy, increased antioxidant ability and extended product shelf life. As this technology continues to be used at an increasingly high rate because of the incorporation of nanoparticles such as ZnO, Ag, and TiO_2_, there are also many associated risks with regard to migration, toxicology, regulatory approval and consumer acceptance (Table 4). These include primary concerns regarding the migration of nanoparticles and the possible release of dissolved forms into packaged foods. The migration of nanoparticles from food contact layers can depend on a variety of parameters, such as the type of polymer matrix, the characteristics of the nanoparticle, the storage conditions (temperature) and duration, and the physical properties of the packaged food itself (i.e., pH and moisture content) [191]. For example, ZnO nanoparticles may partially dissolve in aqueous media and release soluble Zn^2+^ ions, contributing to antimicrobial action; however, the necessary cautionary measures must be taken to evaluate their safety implications on the basis of dose-toxicity relationships [192].

From a toxicological standpoint, the biological impact of nanoparticles is heavily influenced by their chemical nature, size, concentration and exposure conditions. For example, some types of nanoparticles can generate ROS and thus trigger oxidative stress, which results in various forms of cellular injury, such as lipid peroxidation, DNA injury and inflammation [189]. Owing to their broad-spectrum antimicrobial activity against harmful microorganisms, ZnO nanoparticles have been extensively investigated for use in food packaging. However, owing to their potential toxicity to mammalian cells (via mechanisms such as oxidative stress, mitochondrial dysfunction and cell-membrane disruption), these effects may appear at higher concentrations than the amounts typically present in food products [193]. Although reported migration levels from nanocomposite food-packaging materials are generally within regulatory limits in many cases, uncertainties remain regarding long-term exposure, nanoparticle transformation during storage, and the safety assessment of emerging nanomaterials. Therefore, comprehensive migration studies, standardized toxicity evaluations, and clear regulatory frameworks are needed to support the safe application of nanocomposite packaging systems.

**Table 4:** Safety and regulatory considerations for nanocomposite films in meat packaging, risks and mitigation strategies.

Aspect	Key Concern	Main Influencing Factors	Implications in Meat Packaging	Mitigation Strategies	References
Nanoparticle migration	Migration of ZnO, Ag, and TiO_2_ nanoparticles or ions into meat	Particle size, polymer interaction, storage temperature, pH, fat and moisture content	Increased migration may occur in acidic and high-moisture meat products	Improve polymer–nanoparticle compatibility; use coated nanoparticles; apply multilayer packaging	[191,192]
Toxicological effects	Possible oxidative stress, cytotoxicity, and inflammatory responses	ROS generation, nanoparticle concentration, exposure duration, and particle size	Long-term health effects remain insufficiently understood	Use minimum effective concentration; conduct long-term in vivo studies; prefer biodegradable nanofillers	[194]
Food safety assessment	Limited information on chronic exposure and nanoparticle behavior in foods	Interaction with proteins, lipids, and food matrices	Difficulties in establishing universal safety limits	Perform migration and toxicological studies under realistic meat storage conditions	[192,195]
Regulatory challenges	Lack of harmonized nano specific regulations	Differences among regional regulatory frameworks	Delays commercialization and industrial adoption	Develop standardized testing protocols and international guidelines	[192]
Consumer acceptance	Public concern regarding nanotechnology in food packaging	Risk perception, labeling transparency, and awareness	Reduced market acceptance despite functional benefits	Improve safety communication and transparent labeling	[194,196]
Environmental concerns	Potential nanoparticle release during degradation and disposal	Biodegradability, persistence, and waste management practices	Possible environmental accumulation and ecological impact	Use eco-friendly biopolymers and low-toxicity nanomaterials	[196]

However, there is still no clear and comprehensive regulatory framework concerning the nanomaterials used in food contact materials. For example, while the European Union has been rather cautious regarding the use of nanomaterials by imposing specific migration limits, other regions still lack a well-regulated system. According to the EFSA, all engineered nanomaterials need proper toxicological risk assessment [197]. The same cannot be said of other developing countries that do not have proper frameworks for assessing and commercializing the use of nanomaterials. The FDA and WHO suggest that cases should be assessed individually, taking into consideration their migration behavior and toxicity.

Acceptance by the consumer is also a significant challenge in the commercialization process. Even with all the benefits offered by nanopackaging, many consumers have associated the term “nano” with health hazards and uncertainties about its long-term impact [198]. However, the acceptance rate increases dramatically when items are tagged as “natural” or “biobased” or when information about their safety is made available. Therefore, public communication and regulatory clarity are essential for the successful market adoption of nanocomposite packaging systems.

## 6. Sustainability and Environmental Impact

### 6.1. Biodegradability and Compostability

**Figure 6:**
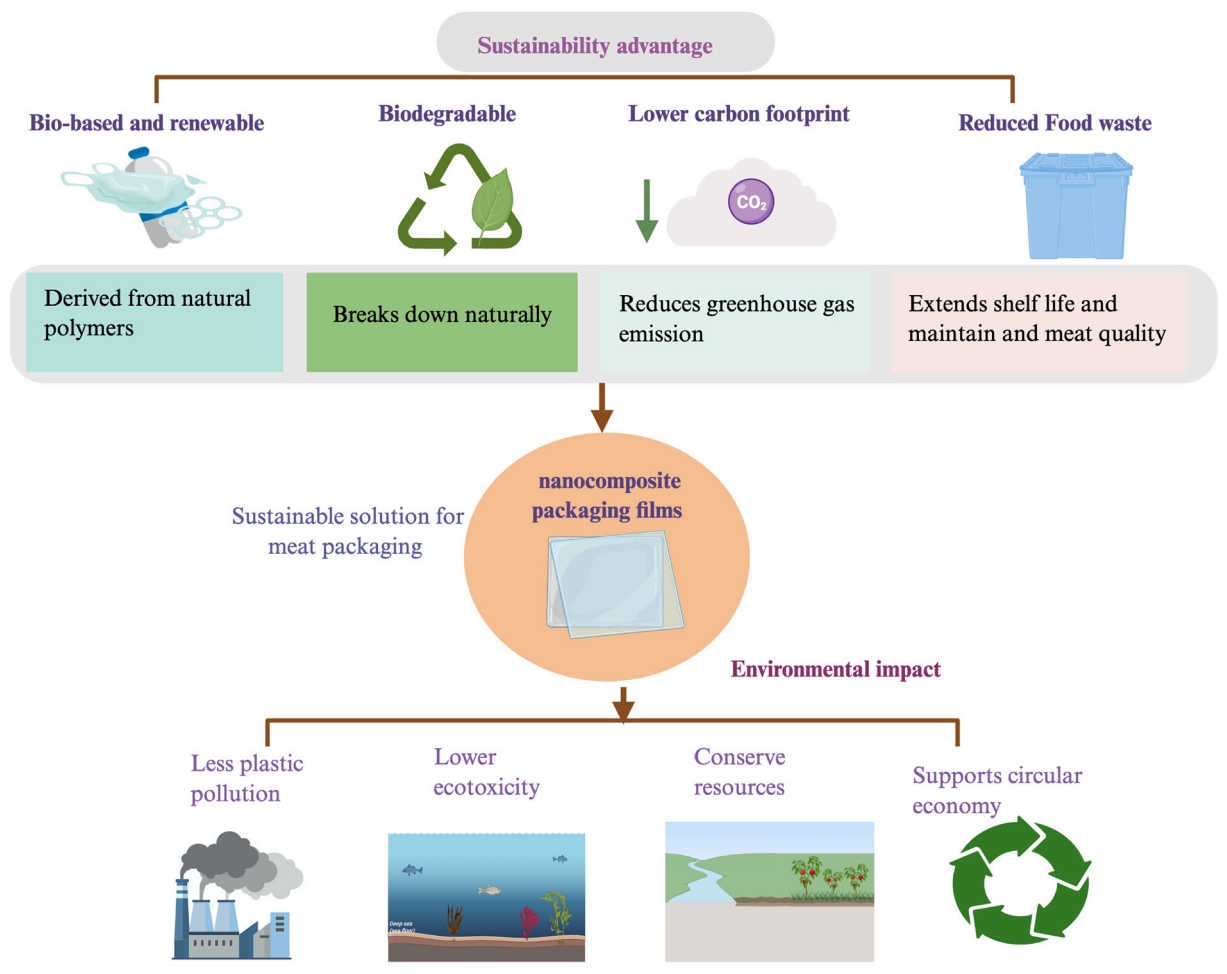
Sustainability and Environmental Impact Pathways of Nanocomposite Films.

Biodegradability and compostability are important sustainability indicators for biopolymer-based nanocomposite films and coatings used in meat packaging (Figure 6). These materials are proposed as alternatives to petroleum-based plastics because polymers such as starch, cellulose, chitosan, alginate, gelatin, soy protein, and whey protein (Figure 2) can be broken down by microorganisms under suitable environmental conditions [199]. However, biodegradability should not be treated as an automatic guarantee of environmental safety. It depends on the polymer type, film thickness, crystallinity, hydrophilicity, cross-linking, nanofiller type, nanofiller concentration, temperature, moisture, oxygen availability, and microbial activity.

Recent studies have shown that some biopolymer nanocomposite films can degrade strongly under soil-burial conditions. Rayhan et al. [200] reported that carboxymethyl cellulose/ZnO nanocomposite films derived from *Zea mays* husk and reinforced with *Ananas comosus* peel extract resulted in more than 96% weight loss after three weeks of soil burial. This indicates strong biodegradability under the tested conditions. Similarly, Vyas et al. [201] reported that ZnO-embedded carboxymethyl cellulose films synthesized from sugarcane bagasse decomposed by 71.43% to 100% within seven days under ambient soil-burial conditions. These findings support the potential of agro-waste-derived CMC films as biodegradable packaging materials.

However, other studies have shown that nanoparticles can slow biodegradation. Heryanto et al. [202] reported that a control smart packaging film degraded by 68.15% after 15 days, whereas ZnO-reinforced films degraded more slowly: 56.42% for 6% ZnO and 49.87% for 10% ZnO. This shows that ZnO can improve film function but reduce the degradation rate, probably by increasing water resistance and reducing microbial access to the film matrix.

The same trend was found for the starch/chitosan nanoparticle films. Othman et al. [203] reported that starch films containing chitosan nanoparticles biodegraded more slowly than neat starch films did. When the CNP concentration increased from 10% to 25%, the degradation rate decreased by 46% in the compost soil and 44% in the planted soil. The neat starch films were fully degraded after eight days in the compost soil, whereas the nanocomposite films degraded more slowly because the added nanoparticles made the structure more compact and resistant to water penetration.

A biodegradable film may breakdown over time, but a compostable film must disintegrate under composting conditions within an acceptable period and should not leave harmful residues [204]. This distinction is important for nanocomposite packaging because the biopolymer matrix may degrade, whereas nanofillers such as ZnO, TiO_2_, Ag nanoparticles, nanoclays, and carbon-based materials may remain in the soil or compost. Overall, biopolymer-based nanocomposite films can reduce the dependence on petroleum-based plastics and may be highly degradable under suitable soil or composting conditions. Nanofillers can improve mechanical strength, barrier properties, and antimicrobial activity, but they may also slow degradation or leave residues after disposal. For meat packaging, the key challenge is to balance storage performance with safe end-of-life behavior [205].

### 6.2. Life Cycle Assessment (Lca)

The need for life cycle assessments (LCAs) is to determine whether nanocomposite films and coatings can significantly reduce the overall environmental impacts associated with meat packaging. A material cannot simply be deemed sustainable by being biobased, biodegradable, or compostable; rather, its value in terms of sustainability will depend upon the entire system, including raw material extraction/processing, manufacturing of the film/coating, packaging performance and ability to extend shelf life, reduced food waste, transport, and end-of-life treatment [206].

For meat packaging, the functional unit is very important. If the functional unit is only based on the weight of the packaging material, the lightest package may appear to be the most sustainable. However, this approach can be misleading because the real function of meat packaging is to deliver safe and acceptable meat to consumers. Casson et al. [207] used one package containing 500 g of sliced beef as the functional unit and compared it with overwrap, high-oxygen modified atmosphere packaging, and vacuum skin packaging. When only packaging impact was considered, overwrap had the best environmental performance in most impact categories. However, when food waste caused by a shorter shelf life was included, vacuum skin packaging became the best environmental option because it had the longest shelf life. This finding shows that less packaging is not always more sustainable for meat products. Similarly, Tetteh et al. [208] clearly described this phenomenon in a chicken meat packaging LCA. Extending the shelf life of chicken meat from 6 to 15 days via modified-atmosphere packaging reduced food waste from 47% to 15% and lowered the impact of climate change by 78%. This result shows that packaging with greater material input may still be environmentally preferable if it prevents greater meat losses. Similar findings were reported for pork belly packaging. Marcos et al. [209] compared two modified-atmosphere packaging systems and vacuum skin packaging for sliced pork belly with low, medium, and high fat contents. Compared with modified-atmosphere packaging, vacuum skin packaging has the longest shelf life, 13–14 days, with a lower purge and reduced fat content and color oxidation. The LCA also revealed that vacuum skin packaging was generally the most environmentally favorable option for low- and medium-fat pork belly, although the best option for high-fat pork belly depended on the impact category.

Recent beef packaging research also supports the need to assess both the environmental impact and product quality. Mukta et al. [210] evaluated recycled, biodegradable, biobased, and blended film materials for ground beef packaging via cradle-to-gate LCA in accordance with [211]. This study revealed that, compared with traditional materials, PCR-LDPE, PLA, and PLA/PBAT blends presented lower global warming potential and fossil fuel depletion. In addition, the color retention of the PLA/PBAT blends improved during retail display, whereas the color retention of the PCR-LDPE blends was similar to that of PVC. This finding indicates that alternative packaging materials should be judged by both environmental indicators and meat-quality performance. These studies highlight an important lesson for the packaging of nanocomposite meat; sustainability is conditional. Nanofillers may improve mechanical strength, oxygen and water vapor barrier properties, antimicrobial activity, and antioxidant performance, but they may also increase production complexity and the environmental burden. Therefore, a nanocomposite film is environmentally justified only when its improved performance leads to clear benefits, such as longer shelf life, reduced spoilage, lower meat rejection, or reduced material use.

## 7. Challenges and Limitations

Although sustainable nanocomposite films and coatings have demonstrated considerable potential for extending shelf life and improving the safety of meat and meat products through enhanced antimicrobial activity, antioxidant properties, and barrier performance, several scientific, technological, regulatory, and commercial challenges continue to hinder their large-scale implementation (Table 4). One of the most significant technological challenges in the production of these composite films is controlling for a uniform distribution of nanoparticles. When nanoparticles are dispersed within polymer matrices during film formation, they have a high surface area, which causes them to bind together and form aggregates. These aggregates result in heterogeneous microstructures that significantly diminish the mechanical strength and barrier properties to gases and water as well as decrease the optical clarity and antimicrobial efficacy of the material. The difficulty of creating uniform nanoparticle distributions increases when moving from the laboratory scale to the industrial scale because it is much easier to maintain consistent nanoparticle dispersions at lower scales [212]. Many of the different film fabrication techniques that have been developed, such as solvent casting, electrospinning, melt or solution extrusion, and layer-by-layer assembly, have shown promising results at the laboratory scale, but few have proven feasible at larger scales because of the extremely high cost associated with producing films, slow processing speeds, the need to recover solvents, and the lack of batch-to-batch reproducibility [14,213].

The long-term active nanocomposite packaging stability is also a major issue. In general, meat products are stored under refrigeration at high humidity. These effects can lead to increased swelling of polymers, aggregation of nanoparticles and early release or degradation of active ingredients, including essential oils and metal nanoparticles. The ability of active packaging to protect against microbes, antioxidants and barrier properties can deteriorate over time because of exposure to these conditions, which can cause the long-term performance of nanocomposites to deteriorate throughout their shelf life [11,214]. Therefore, future studies should look at controlling the delivery of active agents via controlled-release technology, encapsulating nanoparticles via nanoencapsulation technologies, modifying the multilayered structure of the film and modifying the surface chemistry of the nanoparticles to provide longer-term protection from degradation.

Food safety remains one of the most important considerations for nanocomposite packaging because nanoparticles or their ionic degradation products may migrate into food during storage. However, the migration behavior differs considerably among nanomaterials. Zinc oxide (ZnO), one of the most extensively studied antimicrobial nanomaterials, is generally recognized as safe (GRAS) by the U.S. Food and Drug Administration (FDA). Furthermore, the European Food Safety Authority (EFSA) concluded that ZnO incorporated into food-contact materials does not migrate in nanoparticulate form under the intended conditions of use. Instead, ZnO gradually dissolves, releasing zinc ions (Zn^2+^) [11], and its use is regulated under Commission Regulation (EU) No. 10/2011, which establishes a specific migration limit of 5 mg Zn/kg food and an overall tolerable upper intake level of 25 mg Zn/person/day [189]. Nevertheless, migration behavior may still be influenced by factors such as food composition, pH, storage temperature, contact time, and packaging formulation. Consequently, comprehensive migration studies and long-term toxicological assessments remain essential, particularly for other inorganic nanomaterials, such as silver nanoparticles, for which uncertainties regarding chronic exposure, bioaccumulation, and potential toxicity have not yet been fully resolved [215,216]. Another important limitation is the absence of globally harmonized regulatory frameworks governing the use of nanomaterials in food-contact applications. Although regulatory agencies such as the U.S. FDA and EFSA have established guidance for evaluating selected nanomaterials, safety assessment procedures, migration testing protocols, labeling requirements, and authorization criteria vary considerably among countries. These regulatory inconsistencies create uncertainty for manufacturers and may delay the commercialization of innovative nanocomposite packaging systems [217].

Consumer acceptance is another major obstacle to widespread usage. Many consumers continue to express concerns regarding potential adverse impacts on their health and the environment associated with the consumption of foods packaged in materials containing nanomaterials. As such, it will be important to improve public understanding via open and honest communications concerning the perceived risks of nanotechnology-based food packaging, the standardization of label information and the development of scientifically supported evaluations to determine whether these products are safe. For nanocomposites to achieve widespread commercial acceptance, improving the public’s trust in this emerging technology is necessary [43,218]. Finally, there are numerous gaps in knowledge related to how the release of engineered nanomaterials from nanocomposite packages occurs throughout all phases of their life cycle, including production, use, reuse/recycling, and ultimate disposal. The lack of understanding regarding how engineered nanoparticles move and interact with microorganisms, plant species and/or other aquatic organisms within both terrestrial and aquatic ecosystems can result in unforeseen ecological consequences that could occur over extended periods. To address these issues, comprehensive life cycle analyses of nanocomposite packaging, standardized ecotoxicity evaluations of various nanocomposites, and the implementation of viable and sustainable methods to manage the final disposition of nanocomposite packaging waste are crucial [15,17,219]. Overall, while sustainable nanocomposite films and coatings offer substantial opportunities for improving meat preservation, their successful commercialization will require continued advances in scalable manufacturing, controlled nanoparticle dispersion, long-term safety assessment, harmonized regulatory approval, consumer acceptance, and environmental sustainability. Addressing these multidisciplinary challenges will accelerate the transition of nanocomposite packaging from laboratory-scale innovation to widespread industrial application.

## 8. Future Trends and Research Directions

Future research on antimicrobial biodegradable nanocomposite packaging should focus on bridging the gap between laboratory-scale achievements and practical food-packaging applications. Although nanocomposite systems based on polysaccharides, proteins, and bioactive compounds have demonstrated improved antimicrobial activity, antioxidant capacity, and barrier performance, their effectiveness strongly depends on the polymer structure, nanofiller characteristics, active-compound interactions, processing conditions, and complexity of the meat matrices. Therefore, future development should prioritize multifunctionality, safety, scalability, and industrial applicability.

A major research direction is the development of safe-by-design nanocomposites, where the nanoparticle size, morphology, surface chemistry, concentration, and compatibility with polymer matrices are carefully optimized. While nanofillers such as ZnO, TiO_2_, Ag, silica, and nanoclays can enhance antimicrobial and barrier properties, excessive loading may lead to aggregation, structural defects, reduced mechanical performance, and increased migration concerns [220,221]. Future safety evaluations should therefore consider nanoparticle transformation, dissolution, aggregation, and interactions with food components under realistic storage conditions rather than relying only on total migration measurements. Standardized migration protocols and long-term toxicological assessments are essential for regulatory acceptance.

The sustainable development of these materials will increasingly rely on renewable and waste-derived nanofillers. Seafood-processing byproducts, particularly shrimp and crustacean wastes, provide valuable sources of chitin, chitosan, and chitin nanofibers with antimicrobial and reinforcing properties [222]. These materials offer opportunities to integrate waste valorization with biodegradable packaging development. However, sustainability claims should be validated through life-cycle assessment, considering extraction methods, chemical consumption, energy requirements, and end-of-life behavior [223].

Future meat-packaging systems are expected to advance toward multifunctional active and intelligent platforms that combine antimicrobial protection, oxidation control, barrier improvement, and freshness monitoring. The integration of nanosensors and indicators may enable real-time detection of spoilage-related changes, including pH variation, volatile compounds, microbial metabolites, and temperature abuse. However, improvements in sensor stability, food-contact safety, manufacturing compatibility, and cost reduction are needed before widespread commercial application.

Controlled-release strategies, including encapsulation, nanoemulsion, nanofibers, and multilayer structures, represent promising approaches for improving antimicrobial efficiency while reducing undesirable sensory effects. Nevertheless, release behavior remains difficult to predict because it is influenced by the polymer structure, environmental conditions, active-compound characteristics, and food composition. Future research should therefore develop mathematical and computational models to predict antimicrobial release and optimize active-compound delivery under different meat-storage scenarios.

Advanced analytical and predictive approaches will become increasingly important for rational packaging design. The integration of spectroscopy, microscopy, thermal analysis, and molecular-level characterization with artificial intelligence (AI), machine learning, and predictive modeling can establish relationships among material composition, nanofiller dispersion, antimicrobial activity, migration behavior, and shelf life extension. Predictive shelf life models and digital twin approaches that integrate material properties, microbial growth kinetics, and storage conditions may further enable real-time optimization of packaging performance throughout processing, transportation, and distribution.

Despite rapid progress, several research gaps remain. Differences in microbial models, storage conditions, packaging configurations, and evaluation methods often limit comparisons among studies. Future investigations should adopt standardized testing approaches using realistic meat products, mixed microbial populations, and commercial packaging environments to better predict performance during actual storage and distribution. Greater attention should also be given to industrial-scale processing, including extrusion, coating, printing, and thermoforming, together with technoeconomic evaluation.

Finally, sustainable antimicrobial biodegradable packaging should follow circular economy principles by promoting renewable resources, waste valorization, reduced material consumption, and environmentally responsible disposal strategies. However, biodegradability alone should not be considered sufficient evidence of sustainability; complete packaging systems should be evaluated through life-cycle assessment and environmental fate studies. Overall, the future success of antimicrobial biodegradable nanocomposites will depend on achieving a balance among preservation performance, safety, affordability, scalability, consumer acceptance, and environmental sustainability.

## 9. Conclusions

Sustainable nanocomposite films and coatings have strong potential to improve meat preservation while reducing the dependence on conventional petroleum-based packaging. However, the available evidence indicates that no single formulation is suitable for all meat products. Packaging performance depends on the dominant spoilage mechanism of the product, the characteristics of the polymer matrix and nanofiller, compatibility and dispersion, active agent release behavior, and storage conditions. Therefore, future systems should be designed according to the specific preservation needs of fresh meat, poultry, seafood, processed meat, and ready-to-eat products. The benefits of nanocomposite packaging also involve important trade-offs. Increasing the nanofiller concentration may improve the mechanical, barrier, antimicrobial, or antioxidant performance, but excessive loading can cause aggregation, reduced flexibility, structural defects, sensory changes, and increased migration concerns. Similarly, promising laboratory results cannot be assumed to translate directly to commercial conditions because studies use different formulations, food models, testing methods, and storage environments.

Successful commercialization will require more than improved preservation performance. Standardized real-food testing, long-term migration and toxicity assessment, scalable production, regulatory clarity, consumer acceptance, cost evaluation, and life-cycle assessment must be considered together. Future research should therefore prioritize safe-by-design, product-specific formulations that balance functionality, safety, environmental impact, and industrial feasibility. Overall, biodegradable nanocomposite packaging can contribute to longer shelf life, reduced meat waste, and more sustainable packaging, but its practical success depends on closing the gap between laboratory development and reliable commercial application.

## Figures and Tables

**Table 1:** Relationships among major meat spoilage mechanisms, packaging requirements, and nanocomposite-based preservation strategies.

Spoilage Mechanism	Quality Problem in Meat	Packaging Requirement	Nanocomposite Solution	Key References
Microbial growth	Causes off-odor, slime, discoloration, and safety risks due to rapid microbial proliferation.	Inhibit microbial growth and prevent contamination.	Chitosan, ZnO, Ag, TiO_2_, essential oil, and bioactive nanoparticle-based films provide antimicrobial activity.	[36,37,38]
Lipid oxidation	Causes rancidity, off-flavor, color fading, nutrient loss, and reduced shelf life.	Reduce oxygen permeability and oxidative reactions.	Nanoclay, cellulose nanocrystals, ZnO, TiO_2_, and plant-derived antioxidants improve oxygen barrier and antioxidant performance.	[35,39]
Protein oxidation	Reduces tenderness, water-holding capacity, and nutritional quality.	Minimize oxygen exposure and oxidative damage.	Antioxidant-loaded nanocomposite films reduce reactive oxygen species and protect proteins.	[40,41]
Moisture loss	Leads to drip loss, weight loss, surface drying, and reduced juiciness.	Control water vapor transfer and retain moisture.	PLA, cellulose, alginate, lipid, and nanoclay-reinforced films enhance moisture barrier properties.	[36,37,38]
Color deterioration	Causes discoloration and loss of visual appeal due to pigment oxidation.	Control oxygen and light exposure.	TiO_2_, ZnO, nanoclay, and antioxidant additives maintain color stability.	[42,43]
Sensory deterioration	Reduces odor, flavor, texture, appearance, and overall acceptability.	Provide integrated antimicrobial, antioxidant, and barrier protection.	Multifunctional nanocomposite films preserve overall sensory quality and extend shelf life.	[44,45,46]

## Data Availability

No new data were created or analyzed in this study. Data sharing is not applicable to this article.

## References

[1] Van Dijk M., Morley T., Rau M.L., Saghai Y. (2021). A meta-analysis of projected global food demand and population at risk of hunger for the period 2010–2050. Nat. Food.

[2] Zurek M., Hebinck A., Selomane O. (2022). Climate change and the urgency to transform food systems. Science.

[3] Arsat A., Zulkifly M.I., Abdul Majid M.A., Mohd Zahari M.S. (2024). Strengthening food security: A conceptual scholarly examination of the linkages between food loss and food waste. J. Tour. Hosp. Culin. Arts.

[4] Wani N.R., Rather R.A., Farooq A., Padder S.A., Baba T.R., Sharma S., Mubarak N.M., Khan A.H., Singh P., Ara S. (2024). New insights in food security and environmental sustainability through waste food management. Environ. Sci. Pollut. Res..

[5] Snyder A.B., Martin N., Wiedmann M. (2024). Microbial food spoilage: Impact, causative agents and control strategies. Nat. Rev. Microbiol..

[6] Xie D., Ma H., Xie Q., Guo J., Liu G., Zhang B., Li X., Zhang Q., Cao Q., Li X. (2024). Developing active and intelligent biodegradable packaging from food waste and byproducts: A review of sources, properties, film production methods, and their application in food preservation. Compr. Rev. Food Sci. Food Saf..

[7] Karanth S., Feng S., Patra D., Pradhan A.K. (2023). Linking microbial contamination to food spoilage and food waste: The role of smart packaging, spoilage risk assessments, and date labeling. Front. Microbiol..

[8] Panou A., Karabagias I.K. (2023). Biodegradable packaging materials for foods preservation: Sources, advantages, limitations, and future perspectives. Coatings.

[9] Adak B., Baidya S., Teramoto Y. (2025). Biopolymers and their nanocomposites coated paper-based high barrier and sustainable food packaging materials. Carbohydr. Polym..

[10] Mathew S.S., Jaiswal A.K., Jaiswal S. (2025). A comprehensive review on hydrophobic modification of biopolymer composites for food packaging applications. Food Packag. Shelf Life.

[11] Motelica L., Ficai D., Petrisor G., Oprea O.-C., Trușcǎ R.-D., Ficai A., Andronescu E., Hudita A., Holban A.M. (2024). Antimicrobial hydroxyethyl-cellulose-based composite films with zinc oxide and mesoporous silica loaded with cinnamon essential oil. Pharmaceutics.

[12] Smola-Dmochowska A., Lewicka K., Macyk A., Rychter P., Pamuła E., Dobrzyński P. (2023). Biodegradable polymers and polymer composites with antibacterial properties. Int. J. Mol. Sci..

[13] Yan D., Li Y., Liu Y., Li N., Zhang X., Yan C. (2021). Antimicrobial properties of chitosan and chitosan derivatives in the treatment of enteric infections. Molecules.

[14] Altaf A., Usmani Z., Dar A.H., Dash K.K. (2022). A comprehensive review of polysaccharide-based bionanocomposites for food packaging applications. Discov. Food.

[15] Pattnaik R., Panda S.K., Biswas S., De S., Satahrada S., Kumar S. (2024). Prospects and challenges of nanomaterials in sustainable food preservation and packaging: A review. Discov. Nano.

[16] Zhang J., Zhang J., Zhang L., Qin Z., Wang T. (2025). Review of recent advances in intelligent and antibacterial packaging for meat quality and safety. Foods.

[17] Gupta R.K., Guha P., Srivastav P.P. (2024). Investigating the toxicological effects of nanomaterials in food packaging associated with human health and the environment. J. Hazard. Mater. Lett..

[18] Priyadarshi R., Khan A., Riahi Z., Rhim J.W. (2026). Toxicity and Safety of Nanomaterials in Food Packaging Applications: A Dimension-Based Review. Compr. Rev. Food Sci. Food Saf..

[19] Fabiyi O.A., Ogundele A.V., Akinsipo O.B., Badeji A.A., Silva H.H. (2026). Smart nano-packaging materials for sustainable food preservation and safety. Discov. Food.

[20] Ghanayem H., Elbanna A., Lela R., Fathalla E. (2025). Meat spoilage: A comprehensive review of causes, mechanisms and control strategies. Anim. Health Res. J..

[21] Zhu Y., Wang W., Li M., Zhang J., Ji L., Zhao Z., Zhang R., Cai D., Chen L. (2022). Microbial diversity of meat products under spoilage and its controlling approaches. Front. Nutr..

[22] Fang J., Feng L., Lu H., Zhu J. (2022). Metabolomics reveals spoilage characteristics and interaction of *Pseudomonas lundensis* and *Brochothrix thermosphacta* in refrigerated beef. Food Res. Int..

[23] Zhou Z., Ren F., Huang Q., Cheng H., Cun Y., Ni Y., Wu W., Xu B., Yang Q., Yang L. (2024). Characterization and interactions of spoilage of *Pseudomonas fragi* C6 and *Brochothrix thermosphacta* S5 in chilled pork based on LC-MS/MS and screening of potential spoilage biomarkers. Food Chem..

[24] Zhang W., Zhang Y. (2017). Spoilage microorganisms in meat products. Food Spoilage Microorganisms: Ecology and Control.

[25] Encalada V.M.T. (2025). Assessing Spoilage Risks in Raw Chicken Breast: A Convergence Approach Using Microbiological Analysis, Volatile Profiling, Metagenomics, and Data Analytics for Microbial Growth and Shelf-Life Prediction. Master’s Thesis.

[26] Kang J., Kim B.H., Yoon Y. (2026). Meat Spoilage by Bacteria: Influencing Factors, Volatile Compounds, and Organoleptic Alterations. Food Sci. Anim. Resour..

[27] Amaral A.B., Silva M.V.d., Lannes S.C.d.S. (2018). Lipid oxidation in meat: Mechanisms and protective factors—A review. Food Sci. Technol..

[28] Macho-González A., Garcimartín A., López-Oliva M.E., Bastida S., Benedí J., Ros G., Nieto G., Sánchez-Muniz F.J. (2020). Can meat and meat-products induce oxidative stress?. Antioxidants.

[29] Papuc C., Goran G.V., Predescu C.N., Nicorescu V. (2017). Mechanisms of oxidative processes in meat and toxicity induced by postprandial degradation products: A review. Compr. Rev. Food Sci. Food Saf..

[30] Zhang L., Yang D., Luo R., Luo Y., Hou Y. (2024). Research progress on the mechanism of the impact of myofibrillar protein oxidation on the flavor of meat products. Foods.

[31] Zhu Y., Zhu J., Shi X., Fan M. (2025). Effects of freezing, frozen storage and thawing on the water status, quality, nutrition and digestibility of meat: A review. Food Sci. Nutr..

[32] Pan N., Dong C., Du X., Kong B., Sun J., Xia X. (2021). Effect of freeze-thaw cycles on the quality of quick-frozen pork patty with different fat content by consumer assessment and instrument-based detection. Meat Sci..

[33] Ramanathan R., Denzer M., Kiyimba F., Harr K., Suman S.P., Hunt M., Pfeiffer M., Mafi G.G., Kim Y.H.B. (2023). Role of postmortem bioenergetics in beef colour chemistry. Ital. J. Anim. Sci..

[34] Kondjoyan A., Sicard J., Cucci P., Audonnet F., Elhayel H., Lebert A., Scislowski V. (2022). Predicting the oxidative degradation of raw beef meat during cold storage using numerical simulations and sensors—Prospects for meat and fish foods. Foods.

[35] Domínguez R., Pateiro M., Gagaoua M., Barba F.J., Zhang W., Lorenzo J.M. (2019). A comprehensive review on lipid oxidation in meat and meat products. Antioxidants.

[36] Kowalczyk M., Domaradzki P., Skałecki P., Kaliniak-Dziura A., Stanek P., Teter A., Grenda T., Florek M. (2024). Use of sustainable packaging materials for fresh beef vacuum packaging application and product assessment using physicochemical means. Meat Sci..

[37] Devi L.S., Jaiswal A.K., Jaiswal S. (2024). Lipid incorporated biopolymer based edible films and coatings in food packaging: A review. Curr. Res. Food Sci..

[38] Kumar R., Ghoshal G., Goyal M. (2020). Development and characterization of corn starch based nanocomposite film with AgNPs and plant extract. Mater. Sci. Energy Technol..

[39] Falowo A.B., Fayemi P.O., Muchenje V. (2014). Natural antioxidants against lipid–protein oxidative deterioration in meat and meat products: A review. Food Res. Int..

[40] Domínguez R., Pateiro M., Munekata P.E., Zhang W., Garcia-Oliveira P., Carpena M., Prieto M.A., Bohrer B., Lorenzo J.M. (2021). Protein oxidation in muscle foods: A comprehensive review. Antioxidants.

[41] Hadidi M., Orellana-Palacios J.C., Aghababaei F., Gonzalez-Serrano D.J., Moreno A., Lorenzo J.M. (2022). Plant by-product antioxidants: Control of protein-lipid oxidation in meat and meat products. LWT.

[42] Nalçabasmaz S., Ayhan Z., Cimmino S., Silvestre C., Duraccio D. (2017). Effects of PP-based Nanopackaging on the Overall Quality and Shelf Life of Ready-to-eat Salami. Packag. Technol. Sci..

[43] Ghasemi M.A.G., Hamishehkar H., Javadi A., Homayouni-Rad A., Jafarizadeh-Malmiri H. (2024). Natural-based edible nanocomposite coating for beef meat packaging. Food Chem..

[44] Przybyszewska A., Barbosa C.H., Pires F., Pires J.R.A., Rodrigues C., Galus S., Souza V.G.L., Alves M.M., Santos C.F., Coelhoso I. (2023). Packaging of fresh poultry meat with innovative and sustainable ZnO/pectin bionanocomposite films—A contribution to the bio and circular economy. Coatings.

[45] Arfat Y.A., Benjakul S., Vongkamjan K., Sumpavapol P., Yarnpakdee S. (2015). Shelf-life extension of refrigerated sea bass slices wrapped with fish protein isolate/fish skin gelatin-ZnO nanocomposite film incorporated with basil leaf essential oil. J. Food Sci. Technol..

[46] Mohammadi H., Kamkar A., Misaghi A., Zunabovic-Pichler M., Fatehi S. (2019). Nanocomposite films with CMC, okra mucilage, and ZnO nanoparticles: Extending the shelf-life of chicken breast meat. Food Packag. Shelf Life.

[47] Kandeepan G. (2021). Biodegradable nanocomposite packaging films for meat and meat products: A review. J. Packag. Technol. Res..

[48] Bilal M., Ali M., Shaukat F., Ullah S., Arslan M., Li Z., Xiaobo Z., Wahab M., Guo Y., Sun X. (2026). Plant-Based Biopolymer Composites for Smart and Sustainable Food Packaging: Global Insights, Recent Advances, and Future Research Directions. Food Front..

[49] Antony Jose S., Cowan N., Davidson M., Godina G., Smith I., Xin J., Menezes P.L. (2025). A comprehensive review on cellulose nanofibers, nanomaterials, and composites: Manufacturing, properties, and applications. Nanomaterials.

[50] Li J., Zhang F., Zhong Y., Zhao Y., Gao P., Tian F., Zhang X., Zhou R., Cullen P.J. (2022). Emerging food packaging applications of cellulose nanocomposites: A review. Polymers.

[51] Ghasemlou M., Barrow C.J., Adhikari B. (2024). The future of bioplastics in food packaging: An industrial perspective. Food Packag. Shelf Life.

[52] Karnwal A., Rauf A., Jassim A.Y., Selvaraj M., Al-Tawaha A.R.M.S., Kashyap P., Kumar D., Malik T. (2025). Advanced starch-based films for food packaging: Innovations in sustainability and functional properties. Food Chem. X.

[53] Riofrio A., Alcivar T., Baykara H. (2021). Environmental and economic viability of chitosan production in Guayas-Ecuador: A robust investment and life cycle analysis. ACS Omega.

[54] Vicente F.A., Hren R., Novak U., Čuček L., Likozar B., Vujanović A. (2024). Energy demand distribution and environmental impact assessment of chitosan production from shrimp shells. Renew. Sustain. Energy Rev..

[55] Felicia W.X.L., Rovina K., Zuldin W.H., Suriati L., Huda N., Nurdiani R. (2026). Sustainable Valorization of Alginate, a Review of Green Extraction, Structure–Function Relationships, and Next-Generation Food Applications. Food Bioprocess Technol..

[56] Bugarčić M., Jovanović A., Petrović J., Mišić M., Sokić M., Milivojević M. (2024). Advances in biopolymer production and applications: A comprehensive review of key biomaterials. Metall. Mater. Data.

[57] Dhalsamant K., Dalai A., Pattnaik F., Acharya B. (2025). Biodegradable carbohydrate-based films for packaging agricultural products—A review. Polymers.

[58] Sugumaran K., Ponnusami V. (2017). Review on production, downstream processing and characterization of microbial pullulan. Carbohydr. Polym..

[59] Singh R.S., Kaur N., Singh D., Bajaj B.K., Kennedy J.F. (2022). Downstream processing and structural confirmation of pullulan-A comprehensive review. Int. J. Biol. Macromol..

[60] Insights F.B. (2026). Food Packaging Market Size, Share & Industry Analysis, By Material, Product Type, Packaging Type, Application, End-User, and Regional Forecast, 2026–2034. https://www.fortunebusinessinsights.com/fresh-food-packaging-market-110147.

[61] Bayrak S., Omeroglu M.A. (2026). Biopolymer-Based Food Packaging: Functional Properties, Enhancement Strategies, and Future Perspectives. Food Sci. Nutr..

[62] Verma J., Petru M., Goel S. (2024). Cellulose based materials to accelerate the transition towards sustainability. Ind. Crops Prod..

[63] Bangar S.P., Purewal S.S., Trif M., Maqsood S., Kumar M., Manjunatha V., Rusu A.V. (2021). Functionality and applicability of starch-based films: An eco-friendly approach. Foods.

[64] Khan M.R., Torrieri E., Siroli L., Patrignani F., Allais F., Fadlallah S. (2025). From concept to reality: Current trends in developing tunable biopolymeric materials for food contact applications. Crit. Rev. Food Sci. Nutr..

[65] Tang X., Alavi S., Herald T.J. (2008). Effects of plasticizers on the structure and properties of starch–clay nanocomposite films. Carbohydr. Polym..

[66] Jiang S., Liu C., Wang X., Xiong L., Sun Q. (2016). Physicochemical properties of starch nanocomposite films enhanced by self-assembled potato starch nanoparticles. LWT-Food Sci. Technol..

[67] Ma X., Cheng Y., Qin X., Guo T., Deng J., Liu X. (2017). Hydrophilic modification of cellulose nanocrystals improves the physicochemical properties of cassava starch-based nanocomposite films. LWT.

[68] Dai L., Yu H., Zhang J., Cheng F. (2021). Preparation and characterization of cross-linked starch nanocrystals and self-reinforced starch-based nanocomposite films. Int. J. Biol. Macromol..

[69] Oleyaei S.A., Zahedi Y., Ghanbarzadeh B., Moayedi A.A. (2016). Modification of physicochemical and thermal properties of starch films by incorporation of TiO_2_ nanoparticles. Int. J. Biol. Macromol..

[70] Zhu J., Gao W., Wang B., Kang X., Liu P., Cui B., Abd El-Aty A. (2021). Preparation and evaluation of starch-based extrusion-blown nanocomposite films incorporated with nano-ZnO and nano-SiO_2_. Int. J. Biol. Macromol..

[71] Peighambardoust S.J., Peighambardoust S.H., Pournasir N., Pakdel P.M. (2019). Properties of active starch-based films incorporating a combination of Ag, ZnO and CuO nanoparticles for potential use in food packaging applications. Food Packag. Shelf Life.

[72] Coban O.E., Jamshidi A. (2024). Development of bionanocomposite film based on chia seed mucilage incorporated with ZnO nanoparticles and their application for preserving fresh rainbow trout (*Oncorhynchus mykiss*) fillets. J. Food Meas. Charact..

[73] Suyatma N.E., Gunawan S., Putri R.Y., Tara A., Abbès F., Hastati D.Y., Abbès B. (2023). Active biohybrid nanocomposite films made from chitosan, ZnO nanoparticles, and stearic acid: Optimization study to develop antibacterial films for food packaging application. Materials.

[74] Xiao Y., Liu Y., Kang S., Wang K., Xu H. (2020). Development and evaluation of soy protein isolate-based antibacterial nanocomposite films containing cellulose nanocrystals and zinc oxide nanoparticles. Food Hydrocoll..

[75] Pino P., Ronchetti S., Mollea C., Sangermano M., Onida B., Bosco F. (2021). Whey proteins–zinc oxide bionanocomposite as antibacterial films. Pharmaceutics.

[76] Kanmani P., Rhim J.-W. (2014). Physical, mechanical and antimicrobial properties of gelatin based active nanocomposite films containing AgNPs and nanoclay. Food Hydrocoll..

[77] Zhao Y., Li B., Li C., Xu Y., Luo Y., Liang D., Huang C. (2021). Comprehensive review of polysaccharide-based materials in edible packaging: A sustainable approach. Foods.

[78] Behrooznia Z., Nourmohammadi J. (2024). Polysaccharide-based materials as an eco-friendly alternative in biomedical, environmental, and food packaging. Giant.

[79] Guo S., Guo Y., Yang X., Wang X., Zhang R., Wang S., Li M. (2025). A review on polymer packaging by polysaccharide-based nanocomposite films: Crucial role of nanofillers and future application trends. J. Food Meas. Charact..

[80] Gagaoua M., Bhattacharya T., Lamri M., Oz F., Dib A.L., Oz E., Uysal-Unalan I., Tomasevic I. (2021). Green coating polymers in meat preservation. Coatings.

[81] Arora B., Bhatia R., Attri P. (2018). Bionanocomposites: Green materials for a sustainable future. New Polymer Nanocomposites for Environmental Remediation.

[82] Bahrami B., Behzad T., Salehinik F., Zamani A., Heidarian P. (2021). Incorporation of extracted Mucor Indicus fungus chitin nanofibers into starch biopolymer: Morphological, physical, and mechanical evaluation. Starch-Stärke.

[83] Garcia N.L., Famá L., D’Accorso N.B., Goyanes S. (2015). Biodegradable starch nanocomposites. Eco-Friendly Polymer Nanocomposites: Processing and Properties.

[84] Zarski A., Bajer K., Kapuśniak J. (2021). Review of the most important methods of improving the processing properties of starch toward non-food applications. Polymers.

[85] Singh V., Yadav P., Mishra V. (2020). Recent advances on classification, properties, synthesis, and characterization of nanomaterials. Green Synthesis of Nanomaterials for Bioenergy Applications.

[86] de Souza Coelho C.C., Silva R.B.S., Carvalho C.W.P., Rossi A.L., Teixeira J.A., Freitas-Silva O., Cabral L.M.C. (2020). Cellulose nanocrystals from grape pomace and their use for the development of starch-based nanocomposite films. Int. J. Biol. Macromol..

[87] Santana J., de Carvalho Costa É., Rodrigues P., Correia P., Cruz R.S., Druzian J. (2019). Morphological, barrier, and mechanical properties of cassava starch films reinforced with cellulose and starch nanoparticles. J. Appl. Polym. Sci..

[88] Bagher Abiri A., Baghaei H., Mohammadi Nafchi A. (2023). Preparation and application of active bionanocomposite films based on sago starch reinforced with a combination of TiO_2_ nanoparticles and penganum harmala extract for preserving chicken fillets. Polymers.

[89] Domínguez R., Pateiro M., Munekata P.E., McClements D.J., Lorenzo J.M. (2021). Encapsulation of bioactive phytochemicals in plant-based matrices and application as additives in meat and meat products. Molecules.

[90] Barik M., BhagyaRaj G., Dash K.K., Shams R. (2024). A thorough evaluation of chitosan-based packaging film and coating for food product shelf-life extension. J. Agric. Food Res..

[91] Morin-Crini N., Lichtfouse E., Torri G., Crini G. (2019). Applications of chitosan in food, pharmaceuticals, medicine, cosmetics, agriculture, textiles, pulp and paper, biotechnology, and environmental chemistry. Environ. Chem. Lett..

[92] Irastorza A., Zarandona I., Andonegi M., Guerrero P., de la Caba K. (2021). The versatility of collagen and chitosan: From food to biomedical applications. Food Hydrocoll..

[93] Liu T., Li J., Tang Q., Qiu P., Gou D., Zhao J. (2022). Chitosan-based materials: An overview of potential applications in food packaging. Foods.

[94] Motelica L., Ficai D., Ficai A., Oprea O.C., Kaya D.A., Andronescu E. (2020). Biodegradable antimicrobial food packaging: Trends and perspectives. Foods.

[95] Wang H., Qian J., Ding F. (2018). Emerging chitosan-based films for food packaging applications. J. Agric. Food Chem..

[96] Moalla S., Ammar I., Fauconnier M.-L., Danthine S., Blecker C., Besbes S., Attia H. (2021). Development and characterization of chitosan films carrying Artemisia campestris antioxidants for potential use as active food packaging materials. Int. J. Biol. Macromol..

[97] Flórez M., Guerra-Rodríguez E., Cazón P., Vázquez M. (2022). Chitosan for food packaging: Recent advances in active and intelligent films. Food Hydrocoll..

[98] Souza V.G.L., Rodrigues C., Valente S., Pimenta C., Pires J.R.A., Alves M.M., Santos C.F., Coelhoso I.M., Fernando A.L. (2020). Eco-friendly ZnO/Chitosan bionanocomposites films for packaging of fresh poultry meat. Coatings.

[99] Sasidharan S., Tey L.-H., Djearamane S., Ab Rashid N.K.M., PA R., Rajendran V., Syed A., Wong L.S., Santhanakrishnan V.K., Asirvadam V.S. (2024). Innovative use of chitosan/ZnO NPs bio-nanocomposites for sustainable antimicrobial food packaging of poultry meat. Food Packag. Shelf Life.

[100] Pires J.R., Pereira R., Paz S., Gomes L.A., Souza V.G., Godinho M.H., Duarte M.P., Fernando A.L. (2025). Bioactive Properties of Chitosan/Nanocellulose Films Loaded with Sage Essential Oil. J. Compos. Sci..

[101] Roy S., Priyadarshi R., Rhim J.-W. (2021). Development of multifunctional pullulan/chitosan-based composite films reinforced with ZnO nanoparticles and propolis for meat packaging applications. Foods.

[102] Xiao L., Lapu M., Cui L., Li J., Wang X., Li X., Liu M., Liu D. (2025). Impacts of chitosan/pullulan/carvacrol film on the quality and microbial diversity of refrigerated goat meat. Meat Sci..

[103] Mutlu N., Mor B. (2025). Layer-by-layer chitosan-based films with middle-layer incorporated rosemary oil nanoemulsion for sustained release and minced beef preservation. Eur. Food Res. Technol..

[104] Siddiqui S.A., Sundarsingh A., Bahmid N.A., Nirmal N., Denayer J.F., Karimi K. (2023). A critical review on biodegradable food packaging for meat: Materials, sustainability, regulations, and perspectives in the EU. Compr. Rev. Food Sci. Food Saf..

[105] Sun J., Yang X., Bai Y., Fang Z., Zhang S., Wang X., Yang Y., Guo Y. (2024). Recent advances in cellulose nanofiber modification and characterization and cellulose nanofiber-based films for eco-friendly active food packaging. Foods.

[106] Anjali P.N., Bosco S.J.D., Zainab S., Sunooj K.V. (2024). Cellulose and cellulose derivative-based films. Polysaccharide Based Films for Food Packaging: Fundamentals, Properties and Applications.

[107] Infante-Neta A.A., D’Almeida A.P., Albuquerque T.L.d. (2024). Bacterial cellulose in food packaging: A bibliometric analysis and review of sustainable innovations and prospects. Processes.

[108] Wang F., Hu Z., Ouyang S., Wang S., Liu Y., Li M., Wu Y., Li Z., Qian J., Wu Z. (2024). Application progress of nanocellulose in food packaging: A review. Int. J. Biol. Macromol..

[109] Guo J., Ding K., Li S., Li S., Jin P., Zheng Y., Wu Z. (2025). Polysaccharide-based high barrier food packaging film: Design and application. Crit. Rev. Food Sci. Nutr..

[110] del Rosario Herrera-Rivera M., Torres-Arellanes S.P., Cortés-Martínez C.I., Navarro-Ibarra D.C., Hernández-Sánchez L., Solis-Pomar F., Pérez-Tijerina E., Román-Doval R. (2024). Nanotechnology in food packaging materials: Role and application of nanoparticles. RSC Adv..

[111] Motelica L., Ficai D., Oprea O., Ficai A., Trusca R.-D., Andronescu E., Holban A.M. (2021). Biodegradable alginate films with ZnO nanoparticles and citronella essential oil—A novel antimicrobial structure. Pharmaceutics.

[112] Peng B., Qi X., Qiao L., Lu J., Qian Z., Wu C., Xue Z., Kou X. (2025). Nanocomposite-Enabled Next-Generation food packaging: A comprehensive review on advanced Preparation Methods, functional Properties, preservation Applications, and safety considerations. Foods.

[113] He Y., Ye H.-C., You T.-T., Xu F. (2023). Sustainable and multifunctional cellulose-lignin films with excellent antibacterial and UV-shielding for active food packaging. Food Hydrocoll..

[114] Pirsa S., Shamusi T. (2019). Intelligent and active packaging of chicken thigh meat by conducting nano structure cellulose-polypyrrole-ZnO film. Mater. Sci. Eng. C.

[115] Putra N.R. (2025). Advances in cellulose composite films for sustainable food packaging: A review. Nord. Pulp Pap. Res. J..

[116] Abka-Khajouei R., Tounsi L., Shahabi N., Patel A.K., Abdelkafi S., Michaud P. (2022). Structures, properties and applications of alginates. Mar. Drugs.

[117] Raus R.A., Nawawi W.M.F.W., Nasaruddin R.R. (2021). Alginate and alginate composites for biomedical applications. Asian J. Pharm. Sci..

[118] Yammine P., El Safadi A., Kassab R., El-Nakat H., Obeid P.J., Nasr Z., Tannous T., Sari-Chmayssem N., Mansour A., Chmayssem A. (2025). Types of crosslinkers and their applications in biomaterials and biomembranes. Chemistry.

[119] Abedi-Firoozjah R., Bahramian B., Tavassoli M., Majlesi M., Ghaderi S., Assadpour E., Zhang F., Sadeghi E., Bangar S.P., Jafari S.M. (2025). Alginate-based edible films/coatings/nanofibers in food packaging: A comprehensive review of recent advances. Carbohydr. Polym. Technol. Appl..

[120] Lourenço S.C., Fraqueza M.J., Fernandes M.H., Moldão-Martins M., Alves V.D. (2020). Application of edible alginate films with pineapple peel active compounds on beef meat preservation. Antioxidants.

[121] Alves D., Marques A., Milho C., Costa M.J., Pastrana L.M., Cerqueira M.A., Sillankorva S.M. (2019). Bacteriophage ϕIBB-PF7A loaded on sodium alginate-based films to prevent microbial meat spoilage. Int. J. Food Microbiol..

[122] Yang X., Zhao D., Ge S., Bian P., Xue H., Lang Y. (2023). Alginate-based edible coating with oregano essential oil/β-cyclodextrin inclusion complex for chicken breast preservation. Int. J. Biol. Macromol..

[123] Aziz M.S.A., Salama H.E. (2022). Development of alginate-based edible coatings of optimized UV-barrier properties by response surface methodology for food packaging applications. Int. J. Biol. Macromol..

[124] Priyadarshi R., Kim H.-J., Rhim J.-W. (2021). Effect of sulfur nanoparticles on properties of alginate-based films for active food packaging applications. Food Hydrocoll..

[125] Alves Z., Ferreira N.M., Mendo S., Ferreira P., Nunes C. (2021). Design of alginate-based bionanocomposites with electrical conductivity for active food packaging. Int. J. Mol. Sci..

[126] Ning H., Lu L., Xu J., Lu L., Pan L., Lin Z. (2022). Development of sodium alginate-based antioxidant and antibacterial bioactive films added with IRMOF-3/Carvacrol. Carbohydr. Polym..

[127] Liu M., Zhang X., Wei A., Li H., Zhang H., Zheng L., Xia N., Wang J. (2024). Protein-based active films: Raw materials, functions, and food applications. Compr. Rev. Food Sci. Food Saf..

[128] Roy S., Malik B., Chawla R., Bora S., Ghosh T., Santhosh R., Thakur R., Sarkar P. (2024). Biocompatible film based on protein/polysaccharides combination for food packaging applications: A comprehensive review. Int. J. Biol. Macromol..

[129] Dissanayake T., Trinh B.M., Mekonnen T.H., Sarkar P., Aluko R.E., Bandara N. (2023). Improving properties of canola protein-based nanocomposite films by hydrophobically modified nanocrystalline cellulose. Food Packag. Shelf Life.

[130] Oladzadabbasabadi N., Nafchi A.M., Ariffin F. (2022). Proteins-based bionanocomposites for food packaging applications. Bionanocomposites for Food Packaging Applications.

[131] Mo X., Peng X., Liang X., Fang S., Xie H., Chen J., Meng Y. (2021). Development of antifungal gelatin-based nanocomposite films functionalized with natamycin-loaded zein/casein nanoparticles. Food Hydrocoll..

[132] Amjadi S., Nazari M., Alizadeh S.A., Hamishehkar H. (2020). Multifunctional betanin nanoliposomes-incorporated gelatin/chitosan nanofiber/ZnO nanoparticles nanocomposite film for fresh beef preservation. Meat Sci..

[133] Sayadi M., Amiri S., Radi M. (2022). Active packaging nanocomposite gelatin-based films as a carrier of nano TiO_2_ and cumin essential oil: The effect on quality parameters of fresh chicken. J. Food Meas. Charact..

[134] Chu Q., Lu S., Liu J., Wang J., Guo X. (2025). Development of edible nanocomposite films incorporating cinnamaldehyde-carvacrol for extending the shelf life of ready-to-eat steaks. Food Chem. X.

[135] Sultan M., Ibrahim H., El-Masry H.M., Hassan Y.R. (2024). Antimicrobial gelatin-based films with cinnamaldehyde and ZnO nanoparticles for sustainable food packaging. Sci. Rep..

[136] Kumar K., Ngasotter S., Jakhar J.K., Laxmikant V., Sharma S., Dhruve D., Baraiya R., Xavier K.M., Tameshwar F., Barik P. (2026). Gelatin-based nanocomposites for food packaging: A comprehensive review of sources, nanofillers, mechanisms, fabrication techniques, properties, and applications. Next Mater..

[137] Reji R.E., Mathew C.B., Sabu C.S., Roy S. (2025). A review on gelatin films and coatings for active food packaging: Functional properties and applications. Food Innov. Adv..

[138] Chen L., Ramezan Y., Pourramezan H., Najafi A., Kamkari A., Goksen G., Huang Z., Zhang W. (2025). Soy Protein Isolate (SPI)-Based Films/Coatings for Food Packaging: Research Progress on Properties and Applications. Compr. Rev. Food Sci. Food Saf..

[139] Khosravi Z., Fallah A.A., Abtahi H. (2023). Nanocomposite films based on soy protein Isolate-Montmorillonite nanoclay containing emulsion and nanoemulsion of Zataria Multiflora essential oil for preserving chilled chicken burgers. J. Nutr. Fasting Health.

[140] Ran R., Chen S., Su Y., Wang L., He S., He B., Li C., Wang C., Liu Y. (2022). Preparation of pH-colorimetric films based on soy protein isolate/ZnO nanoparticles and grape-skin red for monitoring pork freshness. Food Control.

[141] Tang S., Wang Z., Li W., Li M., Deng Q., Wang Y., Li C., Chu P.K. (2019). Ecofriendly and biodegradable soybean protein isolate films incorporated with ZnO nanoparticles for food packaging. ACS Appl. Bio Mater..

[142] Huang X., Zhou X., Dai Q., Qin Z. (2021). Antibacterial, antioxidation, UV-blocking, and biodegradable soy protein isolate food packaging film with mangosteen peel extract and ZnO nanoparticles. Nanomaterials.

[143] Schmid M., Müller K. (2019). Whey protein-based packaging films and coatings. Whey Proteins.

[144] Xu Y.p., Wang Y., Zhang T., Mu G.q., Jiang S.j., Zhu X.m., Tuo Y.f., Qian F. (2021). Evaluation of the properties of whey protein films with modifications. J. Food Sci..

[145] Weizman O., Dotan A., Nir Y., Ophir A. (2017). Modified whey protein coatings for improved gas barrier properties of biodegradable films. Polym. Adv. Technol..

[146] Karimi N., Alizadeh A., Almasi H., Hanifian S. (2020). Preparation and characterization of whey protein isolate/polydextrose-based nanocomposite film incorporated with cellulose nanofiber and L. plantarum: A new probiotic active packaging system. LWT.

[147] Hajjar Bargh A., Akhondzadeh Basti A., Khanjari A., Noori N. (2025). Enhancing Chicken Fillets’ Shelf Life: Synergistic Effect of Whey Protein Isolate and *Ziziphora clinopodioides* Essential Oil. Food Sci. Nutr..

[148] Sheerzad S., Khorrami R., Khanjari A., Gandomi H., Basti A.A., Khansavar F. (2024). Improving chicken meat shelf-life: Coating with whey protein isolate, nanochitosan, bacterial nanocellulose, and cinnamon essential oil. LWT.

[149] Kandasamy S., Yoo J., Yun J., Kang H.-B., Seol K.-H., Kim H.-W., Ham J.-S. (2021). Application of whey protein-based edible films and coatings in food industries: An updated overview. Coatings.

[150] Wu Y., Wu H., Hu L. (2024). Recent advances of proteins, polysaccharides and lipids-based edible films/coatings for food packaging applications: A review. Food Biophys..

[151] Zubair M., Pradhan R.A., Arshad M., Ullah A. (2021). Recent advances in lipid derived bio-based materials for food packaging applications. Macromol. Mater. Eng..

[152] Eroglu E., Gallet-Pandellé A., Elagali A., Dunlop S., Brunner M. (2026). Edible Films and Coatings with Compostable End-of-Life Properties as Functional Alternatives to Conventional Flexible Plastics in Food Packaging. Food Rev. Int..

[153] Shen G., Yu G., Wu H., Li S., Hou X., Li M., Li Q., Liu X., Zhou M., Chen A. (2021). Incorporation of lipids into wheat bran cellulose/wheat gluten composite film improves its water resistance properties. Membranes.

[154] da Silva Pereira G.V., da Silva Pereira G.V., Neves E.M.P.X., do Rego J.d.A.R., Brasil D.d.S.B., Lourenço L.d.F.H., Joele M.R.S.P. (2020). Glycerol and fatty acid influences on the rheological and technological properties of composite films from residues of Cynoscion acoupa. Food Biosci..

[155] Kumari M., Kodan S., Khangwal I. (2026). Lipid and Wax: Edible Coating and Films. Trends in Edible Packaging: A sustainable approaches for postharvest management of fruits and vegetables: Sustainable Packaging.

[156] Pandey S., Sekar H., Gundabala V. (2024). Development and characterization of bilayer chitosan/alginate cling film reinforced with essential oil based nanocomposite for red meat preservation. Int. J. Biol. Macromol..

[157] Zhu W., Zhang D., Liu X., Ma T., He J., Dong Q., Din Z.-u., Zhou J., Chen L., Hu Z. (2022). Improving the hydrophobicity and mechanical properties of starch nanofibrous films by electrospinning and cross-linking for food packaging applications. LWT.

[158] Díaz-Montes E. (2022). Polysaccharide-based biodegradable films: An alternative in food packaging. Polysaccharides.

[159] El Sheikha A.F., Allam A.Y., ElObeid T., Basiouny E.A., Abdelaal A.A., Amarowicz R., Oz E., Proestos C., Karrar E., Oz F. (2022). Impact of a carboxymethyl cellulose coating incorporated with an ethanolic propolis extract on the quality criteria of chicken breast meat. Antioxidants.

[160] Sani I.K., Geshlaghi S.P., Pirsa S., Asdagh A. (2021). Composite film based on potato starch/apple peel pectin/ZrO_2_ nanoparticles/microencapsulated *Zataria multiflora* essential oil; investigation of physicochemical properties and use in quail meat packaging. Food Hydrocoll..

[161] Xie Q., Liu X., Zhang Y., Liu G. (2023). Development and characterization of a new potato starch/watermelon peel pectin composite film loaded with TiO_2_ nanoparticles and microencapsulated Lycium barbarum leaf flavonoids and its use in the Tan mutton packaging. Int. J. Biol. Macromol..

[162] Bagher Abiri A., Baghaei H., Huda N., Setyawan H.Y., Mohammadi Nafchi A. (2026). Sago Starch Bionanocomposite Films With *Peganum harmala* and TiO_2_: Enhancing Oxidative Stability and Quality of Chicken Fillets. Food Sci. Nutr..

[163] Xu Y., Rehmani N., Alsubaie L., Kim C., Sismour E., Scales A. (2018). Tapioca starch active nanocomposite films and their antimicrobial effectiveness on ready-to-eat chicken meat. Food Packag. Shelf Life.

[164] Adame M.Y., Wang Y., Shi C., Aziz T., Al-Asmari F., Sameeh M.Y., Cui H., Lin L. (2024). Fortification of pullulan/cassava starch-based edible films incorporated with LC-EO nanoparticles and the application for beef meat preservation. Int. J. Biol. Macromol..

[165] Chandrasekar C.M., Nespoli L., Bellesia T., Ghaani M., Farris S., Romano D. (2024). Fabrication of double layer nanoparticle infused starch-based thermoplastic food packaging system for meat preservation. Int. J. Biol. Macromol..

[166] Ning Y., Shi J., Yu S., Du R., Ge J., Zhao D. (2024). Characterization of exopolysaccharide/starch composite film incorporated with TiO_2_ nanoparticles and its application in chilled meat preservation. Int. J. Biol. Macromol..

[167] Yang T., Cao Y., Chen Y., Zhang X., Wang J., Feng A., Zhang L., Li S., Ma X., Chen R. (2026). Smart pectinase-responsive chitosan nanocomposites packaging film for targeted preservation of refrigerated tilapia. Chem. Eng. J..

[168] Ayoub A.W., Sayed S.M., Hefnawy Y.A., Youssef A.M. (2025). Innovative use of chitosan/PVA/GE/ZnO biofilms based on Sage nanoemulsion for sustainable antimicrobial packaging of chilled chicken meat parts. J. Food Meas. Charact..

[169] Zhang Y.-P., Wang X., Shen Y., Thakur K., Zhang J.-G., Hu F., Wei Z.-J. (2021). Preparation and characterization of bio-nanocomposites film of chitosan and montmorillonite incorporated with ginger essential oil and its application in chilled beef preservation. Antibiotics.

[170] Hosseinzadeh S., Partovi R., Talebi F., Babaei A. (2020). Chitosan/TiO_2_ nanoparticle/Cymbopogon citratus essential oil film as food packaging material: Physico-mechanical properties and its effects on microbial, chemical, and organoleptic quality of minced meat during refrigeration. J. Food Process. Preserv..

[171] Li J., Yang K., Shi X., Li T., Guo L., Guo Y., Wang X. (2026). Chitosan-Based Nanocomposite Films Loaded With Composite Essential Oil Nanoemulsions for Pork Preservation. J. Food Process Eng..

[172] Sarmast E., Shankar S., Salmieri S., Rahmouni S.A., Lacroix M. (2026). Development of an Active Crosslinked Nanocomposite Film Based on Gelatin-Ethyl Cellulose, Trans-Cinnamaldehyde, and Silver Nanoparticles for the Preservation of Sliced Meat. J. Food Sci..

[173] Sani M.A., Ehsani A., Hashemi M. (2017). Whey protein isolate/cellulose nanofibre/TiO_2_ nanoparticle/rosemary essential oil nanocomposite film: Its effect on microbial and sensory quality of lamb meat and growth of common foodborne pathogenic bacteria during refrigeration. Int. J. Food Microbiol..

[174] Khezrian A., Shahbazi Y. (2018). Application of nanocompostie chitosan and carboxymethyl cellulose films containing natural preservative compounds in minced camel’s meat. Int. J. Biol. Macromol..

[175] Roy K.D., Desai S.S., Abishad P., Krishnan R., Vinod V.K., Sruthimol J., Kothakotta A., Barbuddhe S.B., Rawool D.B., Vergis J. (2026). Development of a sodium alginate-silver/zinc oxide–cinnamaldehyde nanocomposite film for coating chicken meat and evaluation of meat safety. RSC Adv..

[176] Alsaeed F., Nama F. (2025). Hammal, Copper Oxide Nanoparticle-Enhanced Sodium Alginate Coating for Chicken Meat Preservation: Antimicrobial Efficacy and Shelf-Life Assessment. J. Mater. Environ. Sci..

[177] Ali M.S., Haq M., Roy V.C., Ho T.C., Park J.-S., Han J.-M., Chun B.-S. (2023). Development of fish gelatin/carrageenan/zein bio-nanocomposite active-films incorporated with turmeric essential oil and their application in chicken meat preservation. Colloids Surf. B Biointerfaces.

[178] Khodanazary A. (2024). Effect of soybean protein isolated/cellulose nanofibers-based nano-biocomposite films enriched with TiO_2_ nanoparticle and tannic acid on the quality properties of refrigerated common carp fillets. J. Food Meas. Charact..

[179] Jamwal V., Mittal A. (2024). Recent progresses in nanocomposite films for food-packaging applications: Synthesis strategies, technological advancements, potential risks and challenges. Food Rev. Int..

[180] Kamkar A., Molaee-Aghaee E., Khanjari A., Akhondzadeh-Basti A., Noudoost B., Shariatifar N., Sani M.A., Soleimani M. (2021). Nanocomposite active packaging based on chitosan biopolymer loaded with nano-liposomal essential oil: Its characterizations and effects on microbial, and chemical properties of refrigerated chicken breast fillet. Int. J. Food Microbiol..

[181] Youssef A.M., El-Sayed H.S., El-Sayed S.M., Fouly M., El-Aziz M.A. (2023). Novel bionanocomposites based on cinnamon nanoemulsion and TiO_2_-NPs for preserving fresh chicken breast fillets. Food Bioprocess Technol..

[182] Sayyari Z., Rabani M., Farahmandfar R., Esmaeilzadeh Kenari R., Mousavi Nadoshan R. (2021). The effect of nanocomposite edible coating enriched with Foeniculum vulgare essential oil on the shelf life of *Oncorhynchus mykiss* fish fillets during the storage. J. Aquat. Food Prod. Technol..

[183] Serrano-León J.S., Bergamaschi K.B., Yoshida C.M., Saldaña E., Selani M.M., Rios-Mera J.D., Alencar S.M., Contreras-Castillo C.J. (2018). Chitosan active films containing agro-industrial residue extracts for shelf life extension of chicken restructured product. Food Res. Int..

[184] Tian B., Liu Y. (2020). Chitosan-based biomaterials: From discovery to food application. Polym. Adv. Technol..

[185] Sobhan A., Muthukumarappan K., Wei L., Zhou R., Tummala H. (2021). Development of a polylactic acid-coated nanocellulose/chitosan-based film indicator for real-time monitoring of beef spoilage. Anal. Methods.

[186] Badawy M.E., Lotfy T.M., Shawir S.M. (2020). Facile synthesis and characterizations of antibacterial and antioxidant of chitosan monoterpene nanoparticles and their applications in preserving minced meat. Int. J. Biol. Macromol..

[187] Li H., Qu S., Ma P., Zhang J., Zhao K., Chen L., Huang Q., Zou G., Tang H. (2023). Effects of chitosan coating combined with thermal treatment on physicochemical properties, bacterial diversity and volatile flavor of braised duck meat during refrigerated storage. Food Res. Int..

[188] Haghighi M., Yazdanpanah S. (2020). Chitosan-based coatings incorporated with cinnamon and tea extracts to extend the fish fillets shelf life: Validation by FTIR spectroscopy technique. J. Food Qual..

[189] Smaoui S., Chérif I., Hlima H.B., Khan M.U., Rebezov M., Thiruvengadam M., Sarkar T., Shariati M.A., Lorenzo J.M. (2023). Zinc oxide nanoparticles in meat packaging: A systematic review of recent literature. Food Packag. Shelf Life.

[190] Alizadeh-Sani M., Tavassoli M., McClements D.J., Hamishehkar H. (2021). Multifunctional halochromic packaging materials: Saffron petal anthocyanin loaded-chitosan nanofiber/methyl cellulose matrices. Food Hydrocoll..

[191] Paidari S., Tahergorabi R., Anari E.S., Nafchi A.M., Zamindar N., Goli M. (2021). Migration of various nanoparticles into food samples: A review. Foods.

[192] Committee E.S. (2021). Guidance on risk assessment of nanomaterials to be applied in the food and feed chain: Human and animal health. EFSA J..

[193] Fernández-Bertólez N., Alba-González A., Touzani A., Ramos-Pan L., Méndez J., Reis A.T., Quelle-Regaldie A., Sánchez L., Folgueira M., Laffon B. (2024). Toxicity of zinc oxide nanoparticles: Cellular and behavioural effects. Chemosphere.

[194] Onyeaka H., Passaretti P., Miri T., Al-Sharify Z.T. (2022). The safety of nanomaterials in food production and packaging. Curr. Res. Food Sci..

[195] Lamri M., Bhattacharya T., Boukid F., Chentir I., Dib A.L., Das D., Djenane D., Gagaoua M. (2021). Nanotechnology as a processing and packaging tool to improve meat quality and safety. Foods.

[196] Sarfraz J., Gulin-Sarfraz T., Nilsen-Nygaard J., Pettersen M.K. (2020). Nanocomposites for food packaging applications: An overview. Nanomaterials.

[197] Ansari M.D.I., Principato L., De Nardo L., Punta C. (2025). Nanomaterials in food packaging: An overview of regulatory frameworks and migration assessment. Food Control.

[198] Zimmermann L., Geueke B., Parkinson L.V., Schür C., Wagner M., Muncke J. (2025). Food contact articles as source of micro-and nanoplastics: A systematic evidence map. npj Sci. Food.

[199] Dirpan A., Ainani A.F., Djalal M. (2023). A review on biopolymer-based biodegradable film for food packaging: Trends over the last decade and future research. Polymers.

[200] Rayhan M.A., Hossain M.M., Rahman T., Mia M.S., Zzaman W. (2026). Development of UV-resistant carboxymethyl cellulose/ZNO-NPs based nanocomposite films derived from *Zea mays* husk and reinforced with *Ananas comosus* peel extract for active food packaging. Carbohydr. Polym. Technol. Appl..

[201] Vyas A., Ng S.-p., Fu T., Anum I. (2025). ZnO-embedded carboxymethyl cellulose bioplastic film synthesized from sugarcane bagasse for packaging applications. Polymers.

[202] Heryanto H., Artasasta M.A., Tahir D., Alomari A., Partini J., Akouibaa A. (2025). Development of a biodegradable smart packaging film with ZnO nanoparticles and anthocyanins for real-time freshness monitoring of shrimps. Food Biosci..

[203] Othman S.H., Ronzi N.D.A., Shapi’i R.A., Dun M., Ariffin S.H., Mohammed M.A.P. (2023). Biodegradability of starch nanocomposite films containing different concentrations of chitosan nanoparticles in compost and planting soils. Coatings.

[204] Afshar S.V., Boldrin A., Christensen T.H., Corami F., Daugaard A.E., Rosso B., Hartmann N.B. (2025). Disintegration of commercial biodegradable plastic products under simulated industrial composting conditions. Sci. Rep..

[205] Mujtaba M., Lipponen J., Ojanen M., Puttonen S., Vaittinen H. (2022). Trends and challenges in the development of bio-based barrier coating materials for paper/cardboard food packaging; a review. Sci. Total Environ..

[206] Kakkar A., Kumar S. (2025). Nanocomposite Films and Coatings for Food Packaging. Nanotechnology in Food Packaging and Preservation.

[207] Casson A., Giovenzana V., Frigerio V., Zambelli M., Beghi R., Pampuri A., Tugnolo A., Merlini A., Colombo L., Limbo S. (2022). Beyond the eco-design of case-ready beef packaging: The relationship between food waste and shelf-life as a key element in life cycle assessment. Food Packag. Shelf Life.

[208] Tetteh H., Balcells M., Sazdovski I., Fullana-i-Palmer P., Margallo M., Aldaco R., Puig R. (2024). Environmental comparison of food-packaging systems: The significance of shelf-life extension. Clean. Environ. Syst..

[209] Marcos B., Quinteiro P., Albano-Gaglio M., Muñoz I., Claret A., Guerrero L., Lloret E., Tejeda J.F., Font-i-Furnols M. (2024). Tailor-made packaging strategies of fresh pork belly with different fat content: Enhancing shelf life while minimizing environmental impact. Meat Sci..

[210] Mukta S., Updegraff K.M., Early M.R., Curtzwiler G.W., Vorst K.L. (2025). Sustainability and “food mileage” of ground beef packaging in the beef industry from post-harvest to retail display. Meat Sci..

[211] (2006). Environmental Management—Life Cycle Assessment—Requirements and Guidelines.

[212] Vasile C., Pamfil D., Râpă M., Darie-Niţă R.N., Mitelut A.C., Popa E.E., Popescu P.A., Draghici M.C., Popa M.E. (2018). Study of the soil burial degradation of some PLA/CS biocomposites. Compos. Part B Eng..

[213] Wang Q., Chen W., Zhu W., McClements D.J., Liu X., Liu F. (2022). A review of multilayer and composite films and coatings for active biodegradable packaging. npj Sci. Food.

[214] Kumar L., Ramakanth D., Akhila K., Gaikwad K.K. (2022). Edible films and coatings for food packaging applications: A review. Environ. Chem. Lett..

[215] Ahari H., Anvar A.A., Ataee M., Naeimabadi M. (2021). Employing nanosilver, nanocopper, and nanoclays in food packaging production: A systematic review. Coatings.

[216] Jeevahan J., Chandrasekaran M. (2019). Nanoedible films for food packaging: A review. J. Mater. Sci..

[217] EFSA Panel on Food Contact Materials E., Aids P., Lambré C., Barat Baviera J.M., Bolognesi C., Chesson A., Cocconcelli P.S., Crebelli R., Gott D.M., Grob K. (2024). Scientific Guidance on the criteria for the evaluation and on the preparation of applications for the safety assessment of post-consumer mechanical PET recycling processes intended to be used for manufacture of materials and articles in contact with food. EFSA J..

[218] do Pilar Gonçalves M., Lorite G.S., Rocha J.M., Miilumaki N., Saavalainen P., Selkala T., Morales-Cid G., Pongrácz E., Rocha C.M., Toth G. (2017). Evaluation of physicochemical/microbial properties and life cycle assessment (LCA) of PLA-based nanocomposite active packaging. LWT.

[219] Ros-Lis J.V., Benitez M. (2024). Recent advances in life cycle assessment of nanomaterials for packaging applications. Nanostructured Materials for Food Packaging Applications.

[220] Zhou X., Zhou X., Zhou L., Jia M., Xiong Y. (2024). Nanofillers in novel food packaging systems and their toxicity issues. Foods.

[221] Dejene B.K. (2024). Advancing natural fiber-reinforced composites through incorporating ZnO nanofillers in the polymeric matrix: A review. J. Nat. Fibers.

[222] Rahaman A., Kumari A., Zeng X.-A., Korin A., Khalifa I., Maqsood S. (2026). Processing of marine derived by-products and their applications: A review. Waste Biomass Valorization.

[223] Zhao Z., Li Y., Du Z. (2022). Seafood waste-based materials for sustainable food packing: From waste to wealth. Sustainability.

